# Molecular phylogeny of the Athetini–Lomechusini–Ecitocharini clade of aleocharine rove beetles (Insecta)

**DOI:** 10.1111/j.1463-6409.2012.00553.x

**Published:** 2012-06-20

**Authors:** Hallvard Elven, Lutz Bachmann, Vladimir I Gusarov

**Affiliations:** Hallvard Elven, National Center for Biosystematics, Natural History Museum, University of OsloPO Box 1172 Blindern, NO-0318 Oslo, Norway; Lutz Bachmann, National Center for Biosystematics, Natural History Museum, University of OsloPO Box 1172 Blindern, NO-0318 Oslo, Norway; Vladimir I. Gusarov, National Center for Biosystematics, Natural History Museum, University of OsloPO Box 1172 Blindern, NO-0318 Oslo, Norway

## Abstract

Elven, E., Bachmann, L. & Gusarov V. I. (2012) Molecular phylogeny of the Athetini–Lomechusini–Ecitocharini clade of aleocharine rove beetles (Insecta). —*Zoologica Scripta*, *41*, 617–636.

It has previously been shown that the Aleocharinae tribes Athetini and Lomechusini form a well-supported clade, which also includes the small Neotropical tribe Ecitocharini. However, neither Athetini nor Lomechusini were recovered as monophyletic. In this study, we addressed the basal phylogenetic relationships among the three tribes using sequence data from (i) a mitochondrial fragment covering the COI, Leu2 and COII genes; (ii) a mitochondrial fragment covering part of the 16S gene, the Leu1 gene and part of the NADH 1 gene; and (iii) a part of the nuclear 18S gene, for 68 Athetini, 33 Lomechusini and 2 Ecitocharini species, plus representatives from 10 other tribes. The athetine subtribe Geostibina was recovered as sister group to the ‘true Lomechusini’, which included the type genus *Lomechusa*. The two clades formed a sister group to the main Athetini clade, which also included Ecitocharini and the ‘false Lomechusini’, a group of New World genera normally placed in Lomechusini. The following changes in classification are proposed: (i) Geostibina Seevers, 1978 is raised to tribal rank, and 13 Athetini genera are placed in Geostibini; (ii) *Ecitodonia* Seevers, 1965; *Ecitopora* Wasmann, 1887, and *Tetradonia* Wasmann, 1894 are moved from Lomechusini to Athetini; (iii) Ecitocharini Seevers, 1965 is placed in synonymy with Athetini; (iv) *Discerota* Mulsant & Rey, 1874 is tentatively included in Oxypodini; (v) *Actocharina* Bernhauer, 1907 is placed in synonymy with *Hydrosmecta* Thomson, 1858.

## Introduction

The rove beetles (Staphylinidae) are one of the two largest families of Coleoptera, comprising (with Scydmaeninae) 32 extant subfamilies and more than 55 000 species (O’Keefe 2005; Thayer 2005; Grebennikov & Newton 2009; Bouchard *et al.* 2011). The Aleocharinae [ca. 1200 genera and 13 000 species (Thayer 2005)] are the largest subfamily within Staphylinidae and also one of the most challenging because of the large number of small and morphologically very similar species. Until now, relatively few phylogenetic studies of aleocharines have been published, whether morphology-based (e.g. Kistner & Jacobson 1990; Steidle & Dettner 1993; Muona 1997; Ahn 2001; Ahn & Ashe 2004; Ashe 2005; Paśnik 2010), molecular (Maus *et al.* 2001; Thomas 2009; Elven *et al.* 2010) or both (Ahn *et al.* 2010). The current classification of the subfamily (e.g. Smetana 2004; Ashe 2007; Bouchard *et al.* 2011) is based mainly on intuitive assessments of morphological characters, and even today, new aleocharine tribes are being erected without phylogenetic justification (e.g. Klimaszewski *et al.* 2010).

In a recent study, Elven *et al.* (2010) addressed the molecular phylogeny of Athetini, the largest of the aleocharine tribes. Their most important discovery was the Athetini–Lomechusini–Ecitocharini clade (further referred to as the ALE clade), a well-supported monophyletic group consisting of the genera traditionally included in three different tribes. Within the ALE clade, the tribes Lomechusini and Ecitocharini were found nested within Athetini. Furthermore, the Lomechusini were not recovered as monophyletic, but formed two separate clades within the ALE clade. These unexpected but statistically well-supported results indicated the need for a tribe-level revision of the whole ALE clade. However, Elven *et al.* (2010) could not do so as their taxon sampling was heavily biased towards their primary focus, the phylogeny of Athetini. The Lomechusini were represented by just three genera and four species, and Ecitocharini were represented by only a single species. A more comprehensive sampling of Lomechusini and Ecitocharini was needed before phylogeny-based changes in the tribe-level classification could be seriously considered. In this study, we specifically address this issue using a broader taxon sample for all three tribes.

The tribe Athetini Casey, 1910 is distributed worldwide and includes more than 170 genera and thousands of species (Newton *et al.* 2000). The genus *Atheta* alone includes 1700 species (A. F. Newton, unpublished data). The majority of athetines are free living, while a few are associated with ants or termites. The tribe is traditionally diagnosed based on a combination of several characters, for example, galea and lacinia of moderate length, tarsal formula 4-5-5, mesocoxae narrowly or moderately separated, mesoventral process narrow, athetine bridge of aedeagus present. However, none of these characters is unique to Athetini. An older available family group name, Callicerini Jakobson, 1908 (non Rondani, 1845), exists for this tribe, but Athetini Casey, 1910 is the name currently in prevailing use (Newton & Thayer 1992). An application has been submitted to the International Commission on Zoological Nomenclature to conserve the name Athetini and suppress Callicerini (Gusarov 2011). The tribe Lomechusini Fleming, 1821 is distributed worldwide, but is most diverse in tropical regions. It includes more than 200 genera and over 2200 species (Hlaváč *et al.* 2011), most of which are associated with ants or termites. The tribe is poorly defined and is traditionally diagnosed by a combination of characters, for example, galea and lacinia significantly elongate, tarsal formula 4-5-5, mesocoxae broadly separated, mesoventral process short and broad, athetine bridge of aedeagus present (Newton *et al.* 2000). Not surprisingly, some genera have repeatedly been moved in and out of Lomechusini (e.g. *Meronera*: cf. Newton *et al.* 2000 and Navarrete-Heredia *et al.* 2002). The Neotropical tribe Ecitocharini Seevers, 1965 includes only 10 genera of derived myrmecophiles associated with army ants of the genus *Eciton* (Kistner & Jacobson 1990). The tribe is diagnosed based on a combination of characters (galea and lacinia of moderate length, tarsal formula 4-5-5, particular glands and gland reservoirs present, mesoventral process narrow, body with distinct polygonal microsculpture) (Kistner & Jacobson 1990).

Phylogenetic relationships involving members of the ALE clade have been studied in several publications. The conclusions and limitations of the studies most relevant for the current study are briefly reviewed here in chronological order.

Kistner & Jacobson (1990) revised and redefined the tribe Ecitocharini and conducted a cladistic analysis of the tribe based on 22 morphological characters. The ingroup included all 10 genera of Ecitocharini, but the outgroup included only a single albeit very large and morphologically diverse genus, *Zyras*. The species used to code the character states for *Zyras* was/were not mentioned. Thus, this study was not designed to test the monophyly of Ecitocharini or to rigorously infer relationships among the ingroup taxa.

Steidle & Dettner (1993) studied the morphology and chemistry of the abdominal tergal gland of 22 aleocharine species from nine tribes (adjusted to current classification). Adding further data from published descriptions of the tergal gland and its products, they constructed a matrix of nine morphological and chemical characters for 27 species from 10 tribes, including six Lomechusini and eight Athetini species. Ecitocharini were not included. In their phylogenetic tree, Athetini, Lomechusini and Aleocharini formed a clade supported by just a single apomorphy (gland reservoir large). Together with Oxypodini (excluding the subtribe Dinardina), they formed a larger clade supported by the presence of two groups of products in the gland secretion. Species belonging to the same tribe/subtribe were lumped into a single terminal taxon, and the study was thus not designed to test the monophyly of the included tribes.

Muona (1997) used 87 binary morphological characters in an analysis of 41 genera from 12 aleocharine tribes aimed at testing the monophyly of Athetini. Ecitocharini were not represented, but the study included the dorylophilous tribe Mimanommatini (listed as Dorylomimini). Unfortunately, the character matrix, phylogenetic trees and other details have not been published, and the main results were only summarized in an abstract for the 15th Meeting of the Willi Hennig Society (Muona 1997). Athetini were recovered as paraphyletic, while Lomechusini were polyphyletic. Six tribes (Myllaenini, Lomechusini, Hoplandriini, Termitohospitini, Termitodiscini and Mimanommatini) formed a clade within Athetini. It is noteworthy that three morphologically highly derived tribes, the myrmecophilous Mimanommatini and the termitophilous Termitohospitini and Termitodiscini, were nested within Athetini.

Ashe (2005) used 27 larval and 133 adult morphological characters to infer a phylogeny of the tachyporine-group subfamilies of Staphylinidae. For the Aleocharinae, 29 genera from 13 tribes were included with the aim to resolve the basal phylogenetic relationships of the subfamily. Athetini were represented by the genera *Atheta*, *Geostiba* and *Pontomalota*, and Lomechusini by *Zyras* and *Drusilla*. Ecitocharini were not included. Lomechusini were recovered as monophyletic, while relationships among the three Athetini genera or between Athetini, Lomechusini and the other tribes of ‘higher’ Aleocharinae (*sensu* Ashe 2005) remained unresolved.

Thomas (2009) published the first molecular phylogeny of Aleocharinae, based on nucleotide sequences of the mitochondrial 12S and 16S RNA genes. The study included eight tribes, with Athetini being represented by three genera. Lomechusini and Ecitocharini were not included. In the resulting trees, the relationships among the athetine genera, or between these and the other included tribes, were not resolved.

Paśnik (2010) published a comprehensive morphological phylogeny of the tribe Tachyusini, usually considered a subtribe of Oxypodini (e.g. Seevers 1978; Smetana 2004). The study was based on 159 adult morphological characters and included 84 species from 14 aleocharine tribes. Athetini *sensu* Paśnik were represented by four genera: *Aloconota*, *Atheta*, *Dinaraea* and *Liogluta*. Lomechusini were represented by four genera: *Amaurodera*, *Drusilla*, *Trachyota* and *Zyras*. Also included was *Meronera*, which has been alternatively placed in Lomechusini (e.g. Newton *et al.* 2000), Oxypodini (Seevers 1978), Tachyusini (Ashe 1985) and Falagriini (Pace 2008). The central result of Paśnik’s study was the recovery of a strongly supported clade, Tachyusini *sensu* Paśnik. Many of the recovered relationships were surprising, and an investigation into the underlying character matrix revealed serious issues with the interpretation and weighting of many important tribal characters, a fact that strongly undermines the validity of Paśnik’s results. One example with a direct bearing on the current study is the athetine bridge in the median lobe of the aedeagus, an important character shared by the Athetini and Lomechusini, which Paśnik erroneously interpreted as missing in Lomechusini, the athetine genus *Thamiaraea*, and in *Meronera*.

Elven *et al.* (2010) presented the first comprehensive molecular phylogeny of Athetini. The study included 80 aleocharine species from 11 tribes. Athetini were represented by 27 genera and 58 species, Lomechusini by three genera and four species, and Ecitocharini by a single species. Also included was the genus *Meronera* (see above). They discovered the ALE clade, consisting of the three tribes Athetini, Lomechusini and Ecitocharini. Within the ALE clade, the athetine genera *Geostiba* and *Earota* formed a sister group to the lomechusine genera *Pella* and *Drusilla*. This clade in turn formed a sister group to the remaining Athetini (referred to as the ‘main Athetini clade’), which also included the tribe Ecitocharini and the lomechusine genus *Myrmedonota*. The (*Geostiba*, *Earota*) clade was an unexpected discovery, but there is at least one tentative morphological synapomorphy for this clade: sensillum *a* of the epipharynx being reduced (Yosii & Sawada 1976: fig. 47B; Gusarov 2002b: fig. 2). This character state is thus a potential synapomorphy of the subtribe Geostibina. The lomechusine genus *Myrmedonota* formed a weakly supported clade with *Meronera* within the main Athetini clade. Although the type genus of Lomechusini was not included in Elven *et al.* (2010), they argued that based on morphology, it seemed more closely related to *Pella* and *Drusilla* than to *Meronera* and *Myrmedonota*. In this study, the name ‘true Lomechusini’ will refer to the clade that includes the type genus *Lomechusa*, while the other clade will be referred to as the ‘false Lomechusini’. *Ecitophya*, the only genus of Ecitocharini included in Elven *et al.* (2010), formed a well-supported clade with the New World genus *Stethusa*, a generalized non-myrmecophile athetine.

The main goal of this study is to firmly resolve the phylogeny of the major lineages of the ALE clade discovered by Elven *et al.* (2010), to revise the tribe-level classification. With taxon sampling expanded in all three ALE tribes, the study aims to test the following hypotheses: (i) Geostibina are a sister clade to the ‘true Lomechusini’, the clade that includes *Lomechusa*; (ii) Geostibina and the ‘true Lomechusini’ form a sister group to the main Athetini clade; (iii) All athetine genera that have sensillum *a* of the epipharynx reduced belong to Geostibina; (iv) Several genera traditionally placed in Lomechusini are not members of the ‘true Lomechusini’ clade, but form a subclade (the ‘false Lomechusini’) within the main Athetini clade; and (v) Ecitocharini are monophyletic, nested within the main Athetini clade, and have *Stethusa* as their sister group.

## Material and methods

### Taxon sampling

Taxa used in this study are listed in Table 1. About half of the sequences were produced for this study, the remaining were taken from Elven *et al.* (2010). The taxa were chosen specifically to address the phylogenetic relationships between Athetini, Lomechusini and Ecitocharini, and to this end, we included a broad representation of the first two tribes. The study includes six athetine genera with reduced sensillum *a* of the epipharynx: *Alevonota*, *Aloconota*, *Callicerus*, *Earota*, *Geostiba* and *Pelioptera*. These genera were hypothesized to belong to a monophyletic Geostibina. We included the type species of the type genera of Athetini, Lomechusini and Geostibina, and the type species of many other genera including *Alevonota*, *Aloconota*, *Callicerus*, *Drusilla*, *Earota* and *Meronera* (indicated in Table 1). The large lomechusine genus *Zyras* is represented by at least three subgenera including the nominotypical subgenus, but not the type species. The tribe Ecitocharini is represented by two genera, but not the type genus.

**Table 1:** List of specimens used in this study

Species name	Tribe	Depository^1^	ZMUN Barcode	Country of origin	GenBank accession numbers		
COI–Leu2–COII	16S–Leu1–NADH1	18S
**Subfamily Tachyporinae**							
*Tachinus proximus* Kraatz, 1855	Tachyporini	ZMUN	10002542	Norway	GQ980859	GQ980968	GQ981067
**Subfamily Aleocharinae (except the ALE clade tribes)**							
*Aleochara moerens* Gyllenhal, 1827 #1	Aleocharini	ZMUN	10002579	Norway	GQ980861	GQ980970	GQ981069
*Aleochara moerens* Gyllenhal, 1827 #2	Aleocharini	ZMUN	10002570	Norway	GQ980862	GQ980971	GQ981070
*Tetrasticta* sp. 1	Aleocharini	ZMUC	10029285	Laos	JN581929	JN581761	JN581846
*Tetrasticta* sp. 2	Aleocharini	ZMUC	10029284	Laos	JN581930	JN581762	JN581847
^*•*^*Cordalia obscura* (Gravenhorst, 1802)	Falagriini	ZMUN	10002651	Greece	GQ980864	GQ980973	GQ981071
*Myrmecopora uvida* (Erichson, 1840) #1	Falagriini	ZMUN	10030945	Greece	JN581919	JN581750	JN581834
*Myrmecopora uvida* (Erichson, 1840) #2	Falagriini	ZMUN	10029111	Greece	JN581920	JN581751	JN581835
*Gymnusa variegata* Kiesenwetter, 1845	Gymnusini	ZMUN	10002641	Romania	GQ980860	GQ980969	GQ981068
^*•*^*Bolitochara pulchra* (Gravenhorst, 1806) #1	Homalotini	ZMUN	10002596	Norway	GQ980866	GQ980974	GQ981073
^*•*^*Bolitochara pulchra* (Gravenhorst, 1806) #2	Homalotini	ZMUN	10002591	Norway	GQ980865	–	GQ981072
*Gyrophaena congrua* Erichson, 1837	Homalotini	ZMUN	10002584	Norway	GQ980867	GQ980975	GQ981074
*Gyrophaena fasciata* (Marsham, 1802) #1	Homalotini	ZMUN	10002585	Norway	GQ980868	GQ980976	GQ981075
*Gyrophaena fasciata* (Marsham, 1802) #2	Homalotini	ZMUN	10002572	Norway	GQ980869	GQ980977	GQ981076
^*•*^*Silusida marginella* (Casey, 1893) #1	Homalotini	ZMUN	10002625	USA	GQ980870	GQ980978	GQ981077
^*•*^*Silusida marginella* (Casey, 1893) #2	Homalotini	ZMUN	10002624	USA	GQ980871	GQ980979	GQ981078
^*••*^*Hoplandria lateralis* (Melsheimer, 1846)	Hoplandriini	ZMUN	10002550	USA	GQ980872	GQ980980	GQ981079
*Myllaena audax* Casey, 1911 #1	Myllaenini	ZMUN	10030903	USA	JN581918	JN581749	JN581833
*Myllaena audax* Casey, 1911 #2	Myllaenini	ZMUN	10002598	USA	GQ980881	–	GQ981088
*Halobrecta* cf. *halensis* Mulsant & Rey, 1873	Oxypodini	ZMUN	10002647	Greece	GQ980966	GQ981065	GQ981172
*Oxypoda praecox* Erichson, 1839	Oxypodini	ZMUN	10002637	Germany	GQ980882	GQ980989	GQ981089
*Thendelecrotona* sp.	Oxypodinini^2^	ZMUN	10002612	South Africa	GQ980967	GQ981066	GQ981173
*Placusa* sp. prope *tachyporoides* (Waltl, 1838)	Placusini	ZMUN	10002541	USA	GQ980883	GQ980990	GQ981090
**Tribe Athetini**							
*Acrotona* sp. prope *assecla* (Casey, 1910)	Athetini	ZMUN	10002544	USA	GQ980884	GQ980991	GQ981091
*Acrotona* sp. prope *austiniana* (Casey, 1910) #1	Athetini	ZMUN	10002543	USA	GQ980885	GQ980992	GQ981092
*Acrotona* sp. prope *austiniana* (Casey, 1910) #2	Athetini	ZMUN	10002547	USA	GQ980886	GQ980993	GQ981093
^*•*^*Actocharina leptotyphloides* (Bernhauer, 1907) #1	Athetini	ZMUN	10002656	Austria	JN581857	JN581688	JN581774
^*•*^*Actocharina leptotyphloides* (Bernhauer, 1907) #2	Athetini	ZMUN	10002657	Austria	JN581858	JN581689	JN581775
*Alevonota egregia* (Rye, 1876) #1	Athetini	ZMUN	10030889	France	JN581859	JN581690	–
*Alevonota egregia* (Rye, 1876) #2	Athetini	ZMUN	10030891	France	JN581860	JN581691	–
^*•*^*Alevonota rufotestacea* (Kraatz, 1856)	Athetini	ZMUN	10030810	France	JN581861	JN581692	–
*Aloconota cambrica* (Wollaston, 1855) #1	Athetini	ZMUN	10030823	Austria	JN581862	JN581693	JN581776
*Aloconota cambrica* (Wollaston, 1855) #2	Athetini	ZMUN	10029295	Austria	JN581863	JN581694	JN581777
*Aloconota currax* (Kraatz, 1856) #1	Athetini	ZMUN	10029300	Austria	JN581864	JN581695	JN581778
*Aloconota currax* (Kraatz, 1856) #2	Athetini	ZMUN	10029301	Austria	JN581865	JN581696	JN581779
*Aloconota gregaria* (Erichson, 1839)	Athetini	ZMUN	10029294	Norway	JN581866	JN581697	JN581780
*Aloconota* sp. #1	Athetini	ZMUN	10030840	Uganda	JN581867	JN581698	JN581781
*Aloconota* sp. #2	Athetini	ZMUN	10030746	Uganda	JN581868	–	JN581782
*Alpinia* sp. prope *alpicola* (Miller, 1859) #1	Athetini	ZMUN	10029305	Romania	JN581869	JN581699	JN581783
*Alpinia* sp. prope *alpicola* (Miller, 1859) #2	Athetini	ZMUN	10002644	Romania	GQ980897	–	GQ981104
^*•*^*Amidobia talpa* (Heer, 1841)	Athetini	ZMUN	10002646	Norway	GQ980898	GQ981002	GQ981105
^*•*^*Amischa analis* (Gravenhorst, 1802) #1	Athetini	ZMUN	10029292	Norway	JN581871	JN581702	JN581786
^*•*^*Amischa analis* (Gravenhorst, 1802) #2	Athetini	ZMUN	10002623	Norway	GQ980895	–	GQ981102
*Amischa nigrofusca* (Stephens, 1832) #1	Athetini	ZMUN	10029304	Norway	JN581872	JN581703	JN581787
*Amischa nigrofusca* (Stephens, 1832) #2	Athetini	ZMUN	10002622	Norway	GQ980896	–	GQ981103
*Atheta* (*Alaobia*) *gagatina* (Baudi di Selve, 1848) #1	Athetini	ZMUN	10002578	Norway	GQ980901	GQ981005	GQ981108
*Atheta* (*Alaobia*) *gagatina* (Baudi di Selve, 1848) #2	Athetini	ZMUN	10002580	Norway	GQ980902	GQ981006	GQ981109
*Atheta* (*Alaobia*) *membranata* G. Benick, 1974	Athetini	ZMUN	10002653	France	GQ980903	GQ981007	–
*Atheta* (*Alaobia*) *scapularis* (C.R.Sahlberg, 1831) #1	Athetini	ZMUN	10030796	France	JN581873	JN581704	–
*Atheta* (*Alaobia*) *scapularis* (C.R.Sahlberg, 1831) #2	Athetini	ZMUN	10030807	France	JN581874	JN581705	–
*Atheta* (*crassicornis*-gr.) *crassicornis* (Fabricius, 1793)	Athetini	ZMUN	10002640	Hungary	GQ980907	GQ981011	GQ981113
*Atheta* (*crassicornis*-gr.) *modesta* (Melsheimer, 1844) #1	Athetini	ZMUN	10002621	USA	GQ980908	GQ981012	GQ981114
*Atheta* (*crassicornis*-gr.) *modesta* (Melsheimer, 1844) #2	Athetini	ZMUN	10002620	USA	GQ980909	GQ981013	GQ981115
*Atheta* (*Datomicra*) *celata* (Erichson, 1837) #1	Athetini	ZMUN	10002560	Norway	GQ980910	GQ981014	GQ981116
*Atheta* (*Datomicra*) *celata* (Erichson, 1837) #2	Athetini	ZMUN	10002556	Norway	GQ980911	GQ981015	GQ981117
*Atheta* (*Datomicra*) *dadopora* (Thomson, 1867)	Athetini	ZMUN	10002554	USA	GQ980912	GQ981016	GQ981118
*Atheta* (*Dimetrota*) *aeneipennis* (Thomson, 1856)	Athetini	ZMUN	10002583	Norway	GQ980913	GQ981017	GQ981119
*Atheta* (*Dimetrota*) *cinnamoptera* (Thomson, 1856)	Athetini	ZMUN	10002582	Norway	GQ980914	GQ981018	GQ981120
*Atheta* (*Dimetrota*) *setigera* (Sharp, 1869)	Athetini	ZMUN	10002639	Romania	GQ980917	GQ981021	GQ981123
*Atheta* (*Dralica*) *vilis* (Erichson, 1837) #1	Athetini	ZMUN	10029114	Belarus	JN581875	JN581706	JN581788
*Atheta* (*Dralica*) *vilis* (Erichson, 1837) #2	Athetini	ZMUN	10002666	Belarus	JN581876	JN581707	JN581789
*Atheta* (*Mycetota*) *laticollis* (Stephens, 1832)	Athetini	ZMUN	10002606	Norway	GQ980920	GQ981024	GQ981126
*Atheta* (*Mycetota*) *pasadenae* Bernhauer, 1906	Athetini	ZMUN	10002642	France	GQ980921	GQ981025	GQ981127
*Atheta* (*Oreostiba*) *bosnica* Ganglbauer, 1895	Athetini	ZMUN	10002638	Romania	GQ980922	GQ981026	GQ981128
*Atheta* (*Oxypodera*) *kenyamontis* Pace, 1986	Athetini	ZMUN	10002586	Kenya	GQ980923	GQ981027	GQ981129
*Atheta* (*Parameotica*) *laticeps* (Thomson, 1856) #1	Athetini	ZMUN	10029115	Belarus	JN581925	JN581755	JN581840
*Atheta* (*Parameotica*) *laticeps* (Thomson, 1856) #2	Athetini	ZMUN	10029116	Belarus	JN581926	JN581756	JN581841
*Atheta* (*ravilla*-gr.) *ravilla* (Erichson, 1839) #1	Athetini	ZMUN	10002548	Norway	GQ980924	GQ981028	GQ981130
*Atheta* (*ravilla*-gr.) *ravilla* (Erichson, 1839) #2	Athetini	ZMUN	10002557	Norway	GQ980925	GQ981029	GQ981131
*Atheta* (s. str.) *contristata* (Kraatz, 1856)	Athetini	ZMUN	10002635	Romania	GQ980926	GQ981030	GQ981132
^*••*^*Atheta* (s. str.) *graminicola* (Gravenhorst, 1806) #1	Athetini	ZMUN	10002561	Norway	GQ980927	GQ981031	GQ981133
^*••*^*Atheta* (s. str.) *graminicola* (Gravenhorst, 1806) #2	Athetini	ZMUN	10002562	Norway	GQ980928	GQ981032	GQ981134
*Atheta* (*Thinobaena*) *vestita* (Gravenhorst, 1806)	Athetini	ZMUN	10002613	Norway	GQ980929	GQ981033	GQ981135
*Atheta* (*vaga*-gr.) *vaga* (Heer, 1839)	Athetini	ZMUN	10002655	France	GQ980930	GQ981034	–
*Atheta* sp. ex gr. *lippa*	Athetini	ZMUN	10002564	USA	GQ980918	GQ981022	GQ981124
*Boreophilia hyperborea* (Brundin, 1940)	Athetini	ZMUN	10002634	Russia	GQ980933	GQ981037	GQ981138
*Boreostiba* sp.	Athetini	ZMUN	10002633	Russia	GQ980934	GQ981038	GQ981139
^*•*^*Brundinia meridionalis* (Mulsant & Rey, 1853)	Athetini	ZMUN	10002667	Ukraine	JN581877	JN581708	JN581790
^*•*^*Callicerus obscurus* Gravenhorst, 1802 #1	Athetini	ZMUN	10030905	Denmark	JN581878	JN581709	JN581791
^*•*^*Callicerus obscurus* Gravenhorst, 1802 #2	Athetini	ZMUN	10030800	Denmark	JN581879	JN581710	JN581792
^*•*^*Dadobia immersa* (Erichson, 1837)	Athetini	ZMUN	10002630	Norway	GQ980953	GQ981055	GQ981159
^*•*^*Dalotia coriaria* (Kraatz, 1856)	Athetini	ZMUN	10002643	France	–	GQ981039	GQ981140
^*•*^*Discerota torrentum* (Kiesenwetter, 1850) #1	Athetini	ZMUN	10029112	France	JN581880	JN581711	JN581793
^*•*^*Discerota torrentum* (Kiesenwetter, 1850) #2	Athetini	ZMUN	10029113	France	JN581881	JN581712	JN581794
*Earota dentata* (Bernhauer, 1906)	Athetini	ZMUN	10002539	USA	GQ980965	GQ981064	GQ981171
^*•*^*Earota reyi* (Kiesenwetter, 1850) #1	Athetini	ZMUN	10029306	France	JN581888	JN581718	JN581799
^*•*^*Earota reyi* (Kiesenwetter, 1850) #2	Athetini	ZMUN	10029307	France	JN581889	JN581719	JN581800
^*•*^*Geostiba* (s. str.) *circellaris* (Gravenhorst, 1806)	Athetini	ZMUN	10002587	Norway	GQ980954	GQ981056	GQ981160
*Geostiba* (*Sibiota*) *bicarinata* Lohse & Smetana, 1988 #1	Athetini	ZMUN	10030875	USA	JN581895	JN581725	JN581807
*Geostiba* (*Sibiota*) *bicarinata* Lohse & Smetana, 1988 #2	Athetini	ZMUN	10030948	USA	JN581896	JN581726	JN581808
*Geostiba* (*Sibiota*) *nubigena* Lohse & Smetana, 1988 #1	Athetini	ZMUN	10030888	USA	JN581897	JN581727	JN581809
*Geostiba* (*Sibiota*) *nubigena* Lohse & Smetana, 1988 #2	Athetini	ZMUN	10030736	USA	JN581898	JN581728	JN581810
*Hydrosmecta eximia* (Sharp, 1869) #1	Athetini	ZMUN	10002661	Austria	JN581899	JN581729	JN581811
*Hydrosmecta eximia* (Sharp, 1869) #2	Athetini	ZMUN	10002659	Austria	JN581900	JN581730	JN581812
*Hydrosmecta gracilicornis* (Erichson, 1839)	Athetini	ZMUN	10002658	Austria	JN581901	JN581731	JN581813
*Hydrosmecta valdieriana* (Scheerpeltz, 1944) #1	Athetini	ZMUN	10002662	Austria	JN581903	JN581733	JN581815
*Hydrosmecta valdieriana* (Scheerpeltz, 1944) #2	Athetini	ZMUN	10002663	Austria	JN581904	JN581734	JN581816
*Hydrosmecta* sp. 1	Athetini	ZMUN	10002650	USA	GQ980955	GQ981057	GQ981161
*Hydrosmecta* sp. 2	Athetini	ZMUN	10002660	Austria	JN581902	JN581732	JN581814
*Liogluta microptera* Thomson, 1867 #1	Athetini	ZMUN	10002600	Czech Republic	GQ980937	GQ981041	GQ981143
*Liogluta microptera* Thomson, 1867 #2	Athetini	ZMUN	10002602	Czech Republic	GQ980936	–	GQ981142
*Liogluta nigropolita* (Bernhauer, 1907)	Athetini	ZMUN	10002636	Russia	GQ980938	–	GQ981144
*Lypoglossa lateralis* (Mannerheim, 1830)	Athetini	ZMUN	10002632	Russia	GQ980887	GQ980994	GQ981094
^*•*^*Lyprocorrhe anceps* (Erichson, 1837)	Athetini	ZMUN	10002649	Norway	GQ980939	GQ981042	GQ981145
^*•*^*Meronera venustula* (Erichson, 1839)	Athetini^3^	ZMUN	10002576	USA	GQ980875	GQ980983	GQ981082
^*•*^*Mocyta fungi* (Gravenhorst, 1806) #1	Athetini	ZMUN	10002588	Germany	GQ980888	GQ980995	GQ981095
^*•*^*Mocyta fungi* (Gravenhorst, 1806) #2	Athetini	ZMUN	10002589	Germany	GQ980889	GQ980996	GQ981096
*Mocyta scopula* (Casey, 1893) #1	Athetini	ZMUN	10002540	USA	GQ980890	GQ980997	GQ981097
*Mocyta scopula* (Casey, 1893) #2	Athetini	ZMUN	10002559	USA	GQ980891	GQ980998	GQ981098
*Pelioptera* sp. prope *micans* (Kraatz, 1857)	Athetini	ZMUC	10030959	Laos	–	JN581758	JN581843
*Philhygra debilis* (Erichson, 1837) #1	Athetini	ZMUN	10002607	Norway	GQ980941	GQ981044	GQ981147
*Philhygra debilis* (Erichson, 1837) #2	Athetini	ZMUN	10002608	Norway	GQ980942	GQ981045	GQ981148
*Philhygra fallaciosa* (Sharp, 1869) #1	Athetini	ZMUN	10002610	Czech Republic	GQ980943	GQ981046	GQ981149
*Philhygra fallaciosa* (Sharp, 1869) #2	Athetini	ZMUN	10002609	Czech Republic	GQ980944	GQ981047	GQ981150
*Philhygra iterans* (Casey, 1910) #1	Athetini	ZMUN	10002595	USA	GQ980945	GQ981048	GQ981151
*Philhygra iterans* (Casey, 1910) #2	Athetini	ZMUN	10002594	USA	GQ980946	GQ981049	GQ981152
^*•*^*Pontomalota opaca* (LeConte, 1863) #1	Athetini	ZMUN	10002577	USA	GQ980956	GQ981058	GQ981162
^*•*^*Pontomalota opaca* (LeConte, 1863) #2	Athetini	ZMUN	10002574	USA	GQ980957	GQ981059	GQ981163
^*•*^*Stethusa dichroa* (Gravenhorst, 1802) #1	Athetini	ZMUN	10002567	USA	GQ980948	GQ981051	GQ981154
^*•*^*Stethusa dichroa* (Gravenhorst, 1802) #2	Athetini	ZMUN	10002568	USA	GQ980949	GQ981052	GQ981155
*Stethusa spuriella* (Casey, 1910) #1	Athetini	ZMUN	10002599	USA	GQ980950	GQ981053	GQ981156
*Stethusa spuriella* (Casey, 1910) #2	Athetini	ZMUN	10002628	USA	GQ980951	GQ981054	GQ981157
^*•*^*Strigota ambigua* (Erichson, 1839) #1	Athetini	ZMUN	10002571	USA	GQ980893	GQ981000	GQ981100
^*•*^*Strigota ambigua* (Erichson, 1839) #2	Athetini	ZMUN	10002575	USA	GQ980894	GQ981001	GQ981101
^*•*^*Tarphiota fucicola* (Mäklin *in* Mannerheim, 1852)	Athetini	ZMUN	10002593	USA	GQ980958	GQ981060	GQ981164
**Tribe Ecitocharini**							
*Ecitomorpha* sp.	Ecitocharini	ZMUN	10002689	Peru	JN581892	JN581722	JN581803
*Ecitophya gracillima* Mann, 1925 #1	Ecitocharini	ZMUN	10002592	Peru	GQ980863	GQ980972	JN581804
*Ecitophya gracillima* Mann, 1925 #2	Ecitocharini	ZMUN	10029164	Peru	JN581893	JN581723	JN581805
**Tribe Lomechusini**							
*Amaurodera yaoana* Pace, 1992 #1	Lomechusini	ZMUC	10030878	Laos	JN581870	JN581700	JN581784
*Amaurodera yaoana* Pace, 1992 #2	Lomechusini	ZMUC	10030812	Laos	–	JN581701	JN581785
^*•*^*Drusilla canaliculata* (Fabricius, 1787) #1	Lomechusini	ZMUN	10002604	Norway	GQ980873	GQ980981	GQ981080
^*•*^*Drusilla canaliculata* (Fabricius, 1787) #2	Lomechusini	ZMUN	10002601	Norway	GQ980874	GQ980982	GQ981081
*Drusilla* sp. 1 #1	Lomechusini	ZMUN	10051252	Thailand	JN581882	JN581713	–
*Drusilla* sp. 1 #2	Lomechusini	ZMUN	10051251	Thailand	JN581883	JN581714	–
*Drusilla* sp. 2 #1	Lomechusini	ZMUC	10051193	Laos	JN581884	JN581715	JN581795
*Drusilla* sp. 2 #2	Lomechusini	ZMUC	10051194	Laos	JN581885	JN581716	JN581796
*Drusilla* sp. prope *khamhengi* Pace, 1984 #1	Lomechusini	ZMUC	10051166	Laos	JN581886	JN581717	JN581797
*Drusilla* sp. prope *khamhengi* Pace, 1984 #2	Lomechusini	ZMUC	10051165	Laos	JN581887	–	JN581798
*Ecitodonia* sp. #1	Lomechusini	ZMUN	10029249	Ecuador	JN581890	JN581720	JN581801
*Ecitodonia* sp. #2	Lomechusini	ZMUN	10029248	Ecuador	JN581891	JN581721	JN581802
*Ecitopora* sp.	Lomechusini	ZMUN	10029251	Ecuador	JN581894	JN581724	JN581806
^*••*^*Lomechusa emarginata* (Paykull, 1789) #1	Lomechusini	ZMUN	10030941	Norway	JN581905	JN581735	JN581817
^*••*^*Lomechusa emarginata* (Paykull, 1789) #2	Lomechusini	ZMUN	10030947	Norway	JN581906	JN581736	JN581818
*Lomechusa pubicollis* Brisout de Barneville, 1860	Lomechusini	ZMUN	10030917	Germany	JN581907	JN581737	JN581819
Lomechusini genus 1	Lomechusini	ZMUN	10029159	Ecuador	JN581908	JN581738	JN581820
Lomechusini genus 2 #1	Lomechusini	ZMUN	10002685	Ecuador	JN581909	JN581739	JN581821
Lomechusini genus 2 #2	Lomechusini	ZMUN	10029161	Ecuador	JN581910	–	JN581822
Lomechusini genus 3 #1	Lomechusini	ZMUN	10029252	Ecuador	–	JN581740	JN581823
Lomechusini genus 3 #2	Lomechusini	ZMUN	10029253	Ecuador	–	JN581741	JN581824
Lomechusini genus 4 #1	Lomechusini	ZMUN	10029256	Ecuador	JN581911	JN581742	JN581825
Lomechusini genus 4 #2	Lomechusini	ZMUN	10029254	Ecuador	JN581912	–	JN581826
Lomechusini genus 5 #1	Lomechusini	ZMUN	10029258	Ecuador	JN581913	JN581743	JN581827
Lomechusini genus 5 #2	Lomechusini	ZMUN	10029257	Ecuador	JN581914	JN581744	JN581828
Lomechusini genus 6	Lomechusini	ZMUN	10029279	Ecuador	JN581915	JN581745	JN581829
Lomechusini genus 7 #1	Lomechusini	ZMUN	10029280	Ecuador	JN581916	JN581746	JN581830
Lomechusini genus 7 #2	Lomechusini	ZMUN	10029282	Ecuador	–	JN581747	JN581831
*Lomechusoides amurensis* (Wasmann, 1897)	Lomechusini	ZMUN	10030868	Russia	JN581917	JN581748	JN581832
*Myrmedonota* sp. #1	Lomechusini	ZMUN	10002615	USA	GQ980876	GQ980984	GQ981083
*Myrmedonota* sp. #2	Lomechusini	ZMUN	10002614	USA	GQ980877	GQ980985	GQ981084
*Orphnebius* (*Mesocephalobius*) sp. 1	Lomechusini	ZMUC	10051237	Laos	JN581921	JN581752	JN581836
*Orphnebius* (*Mesocephalobius*) sp. 2	Lomechusini	ZMUC	10051236	Laos	JN581922	JN581753	JN581837
*Orphnebius* (*Mesocephalobius*) sp. 3	Lomechusini	ZMUN	10051239	Thailand	JN581923	–	JN581838
*Orphnebius* (*Mesocephalobius*) sp. 4	Lomechusini	ZMUN	10051238	Thailand	JN581924	JN581754	JN581839
*Pedinopleurus* sp.	Lomechusini	ZMUN	10051235	Thailand	JN581927	JN581757	JN581842
*Pella caliginosa* (Casey, 1893) #1	Lomechusini	ZMUN	10002617	USA	GQ980878	GQ980986	GQ981085
*Pella caliginosa* (Casey, 1893) #2	Lomechusini	ZMUN	10002616	USA	GQ980879	GQ980987	GQ981086
*Pella humeralis* (Gravenhorst, 1802)	Lomechusini	ZMUN	10002569	Norway	GQ980880	GQ980988	GQ981087
*Tetradonia* sp. 1	Lomechusini	ZMUN	10029163	Ecuador	–	JN581759	JN581844
*Tetradonia* sp. 2	Lomechusini	ZMUN	10029160	Ecuador	JN581928	JN581760	JN581845
*Zyras* (*Glossacantha*) *perdecoratus* Pace, 2005 #1	Lomechusini	ZMUN	10051274	Thailand	JN581932	JN581764	JN581849
*Zyras* (*Glossacantha*) *perdecoratus* Pace, 2005 #2	Lomechusini	ZMUN	10051273	Thailand	JN581933	JN581765	JN581850
*Zyras* (*Glossacantha*) sp. 3 #1	Lomechusini	ZMUN	10051280	Thailand	JN581938	JN581770	JN581853
*Zyras* (*Glossacantha*) sp. 3 #2	Lomechusini	ZMUN	10051279	Thailand	JN581939	JN581771	JN581854
*Zyras* (*Glossacantha*) sp. prope *perdecoratus* Pace, 2005 #1	Lomechusini	ZMUN	10051276	Thailand	JN581940	JN581772	JN581855
*Zyras* (*Glossacantha*) sp. prope *perdecoratus* Pace, 2005 #2	Lomechusini	ZMUN	10051275	Thailand	JN581941	JN581773	JN581856
*Zyras* (*Rhynchodonia*) sp. 2 #1	Lomechusini	ZMUC	10051233	Laos	JN581936	JN581768	JN581851
*Zyras* (*Rhynchodonia*) sp. 2 #2	Lomechusini	ZMUC	10051234	Laos	JN581937	JN581769	JN581852
*Zyras* (s. str.) *collaris* (Paykull, 1800)	Lomechusini	ZMUN	10002669	Abkhasia	JN581931	JN581763	JN581848
*Zyras* sp. 1 #1	Lomechusini	ZMUN	10030963	Thailand	JN581934	JN581766	–
*Zyras* sp. 1 #2	Lomechusini	ZMUN	10030758	Thailand	JN581935	JN581767	–

Type species are marked with a bullet (^*•*^), or two bullets (^••^) if the respective genus is the type of its tribe. Additional label information is provided in Table S1.

1) The specimens listed as deposited at ZMUC will be divided between ZMUC and ZMUN.

2) *Thendelecrotona* was moved from Athetini to Aleocharinae *incertae sedis* in Elven *et al.* (2010), but based on its similarity to the Malagasy genus *Oxypodinus,* we now treat it as a member of Oxypodinini.

3) *Meronera* is listed under Athetini where it was placed by Elven *et al.* (2010). The remaining members of the ‘false Lomechusini’ are listed under Lomechusini.

The tribes Lomechusini and Athetini both have global distributions. The Lomechusini in our data set have a good geographic coverage and include species from the Palaearctic, Nearctic, Neotropical and Oriental regions. The Athetini are mainly represented by Palaearctic and Nearctic species but also include one Oriental and two African species.

Whenever possible, two specimens of each species were sequenced as additional control for misidentifications. The total data set included 180 samples representing 120 species, of which 68 belong to Athetini, 33 to Lomechusini, 2 to Ecitocharini and 16 to nine other aleocharine tribes used as outgroup. *Tachinus proximus* from the subfamily Tachyporinae was included as a more distant outgroup taxon. There was one taxonomic change for the sequences taken from Elven *et al.* (2010): the genus *Thendelecrotona* is here recognized to be a member of the tribe Oxypodinini, based on its similarity to the Malagasy genus *Oxypodinus* in both external characters and the male genitalia.

For most of the Palaearctic and Nearctic specimens, identification to species level was straightforward. For samples from the tropical regions, identification to species level was often impossible and many samples were identified only to genus level. Seven Neotropical species (12 specimens) could not even be assigned to genera, and their initial tribal placement in Lomechusini is based on an assessment of tribal characters, including tarsal formula 4-5-5, mesocoxae relatively broadly separated and mesoventral process short and broad.

Most specimens were collected directly into >96% ethanol and stored at −20 °C prior to processing, but some trap material that had been exposed to high (+20 to +40 °C) temperatures and/or dilution by rain was also included. All specimens used for DNA extraction are labelled as vouchers and deposited at the Natural History Museum, University of Oslo (ZMUN) or the Zoological Museum, Natural History Museum of Denmark (ZMUC). Label information is provided in Table S1.

### Molecular markers

Nucleotide sequences from one nuclear and two mitochondrial regions were targeted. The first mitochondrial region covered most of the cytochrome oxidase 1 and 2 (COI and COII) and the tRNA-Leucine 2 (Leu2) genes. The second mitochondrial region covered the 3′-end of the large ribosomal subunit (16S), the tRNA-Leucine 1 (Leu1) and a small part of the NADH dehydrogenase subunit 1 (NADH1) genes. The nuclear region covered an internal part of the small ribosomal subunit (18S) gene. These markers were used by Elven *et al.* (2010) and have proved suitable for the study of athetine phylogeny.

### DNA extraction, amplification and sequencing

DNA was extracted from the head and prothorax using the Qiagen DNeasy Blood and Tissue Kit (Qiagen, Hilden, Germany) following the manufacturer’s protocol for animal tissue. For the very small specimens of *Actocharina,* the whole body was used. DNA extraction was performed on vacuum-dried samples without prior homogenization. Samples were incubated in lysis buffer for 20–30 h. After extraction, the exoskeletons were retrieved for dry mounting with the rest of the voucher.

The targeted regions were amplified using the primers listed in Table S2. The mitochondrial COI–Leu2–COII region was amplified in three overlapping fragments, while the mitochondrial 16S–Leu1–NADH1 region and the nuclear 18S region were each amplified in single fragments. PCRs were set-up in a 25-μL reaction volume containing 2.5 mm MgCl_2_ (Applied Biosystems, Foster City, CA, USA), 1 × ABI GeneAmp PCR buffer (Applied Biosystems), 0.8 mm GeneAmp dNTPs (Applied Biosystems), 0.5 μm of each primer (MWG-Biotech AG, Ebersberg, Germany), 1 U ABI AmpliTaq DNA Polymerase (Applied Biosystems) and 3 μL template DNA extract. Most reactions also included 1.1 mg dimethyl sulfoxide (Merck, Darmstadt, Germany) to improve PCR performance. When amplifying the 16S–Leu1–NADH1 region, the PCR set-up was adjusted to 2 mm MgCl_2_, 0.96 × PCR buffer, 0.64 mm dNTPs and 0.4 μm of each primer. The amplification profile consisted of an initial denaturation step of 94 °C for 30 s, followed by 30 cycles of 94 °C for 1 min, annealing temperature Ta for 30 s and 72 °C for 2 min, and finally a 10 min extension step at 72 °C. The annealing temperatures are listed in Table S2. For some difficult samples, PCR performance was improved by replacing dimethyl sulfoxide in the reaction mix with 0.4 μg bovine serum albumin (Sigma-Aldrich, Steinheim, Germany), by using alternative primers (listed in Table S2), by lowering the annealing temperature or by using an alternative PCR protocol employing HotStar Taq DNA Polymerase (Qiagen).

PCR products were purified using ExoSAP-IT (USB Corporation, Cleveland, Ohio, USA). If secondary products were detected on a standard agarose gel, the PCR product of appropriate size was cut out from 1% agarose gel and purified using the MN NucleoSpin Extract II gel extraction kit (Macherey-Nagel, Düren, Germany). Purified PCR products were sequenced in both directions using the terminal primers with the ABI BigDye Terminator Cycle Sequencing Kit v3.1 (Applied Biosystems) and analysed on the ABI 3730 DNA Analyzer (Applied Biosystems). All DNA sequencing was outsourced to the ABI-lab, Departments of Biology and of Molecular Biosciences, University of Oslo.

### Sequence alignment and model selection

Alignment of the protein-coding genes was straightforward, as there were virtually no indels. For the RNA-coding genes, published secondary structures from other insect groups were used as a guide for manual alignment in MEGA 4 (Tamura *et al.* 2007). Secondary structures for *Apis mellifera* (Gillespie *et al.* 2006) were used to aid alignment of 16S and 18S, while secondary structures for *Xenos vesparum* (Carapelli *et al.* 2006) were used to aid alignment of Leu1 and Leu2. Loop regions that could not be aligned unambiguously were excluded from the subsequent analyses.

The concatenated alignment was partitioned by codon positions, stems vs. loops, and genomic origin (mitochondrial vs. nuclear) to produce a total of seven partitions (for details, see Elven *et al.* 2010). MrModelTest 2.3. (Nylander 2004) was used to determine a suitable evolutionary model for each partition under the Akaike information criterion.

### Phylogenetic analyses

The partitioned data set was analysed under maximum likelihood (ML) and Bayesian inference. Two analyses were performed with each method; one with all sequences included and one where sequences with more than 20% missing information were excluded (i.e. those lacking data for one of the three targeted regions).

ML analyses were performed in RAxML 7.0.3 (Stamatakis 2006) using the Rapid Bootstrap algorithm (Stamatakis *et al.* 2008) followed by ML optimization. Each analysis was performed with 1000 bootstrap replicates, and every fifth bootstrap tree was used as a starting point for subsequent ML optimization on the original data set. The partitioned data set was analysed using the GTRMIX option (CAT approximation for the bootstrap, followed by final ML optimization under the GTR+Г model). ML optimization was also performed on 100 randomized parsimony trees to test whether this would yield a higher final likelihood score. The tree with the overall highest score was selected as the best tree, and bootstrap values were drawn on this tree.

Prior to the analysis, the rearrangement settings and number of rate categories were optimized following the author’s recommendations (RAxML 7.0.4 Manual). Two alternative rearrangement settings (*i* = auto vs. *i* = 10) were compared by performing ML optimization on 10 randomized parsimony trees with each setting, and choosing the setting resulting in the highest likelihood score for the optimized trees. Four alternative numbers of rate categories (10, 25, 40 and 55) were then compared in the same way using the best rearrangement setting from above. The same 10 starting trees were used for all comparisons. Based on the results of these comparisons, the analysis that included incomplete sequences was performed using *i* = auto, while the analysis using only complete sequences was performed using *i* = 10. Both analyses were performed using 25 rate categories.

Bayesian analyses were performed in MrBayes v3.1.2 (Ronquist & Huelsenbeck 2003) using the GTR+Г model for 3rd codon positions and GTR+I+Г for the other partitions. MrModelTest suggested GTR+I+Г for all partitions, but under this modelling regime, the runs did not converge. Closer inspection of the model parameters showed that several 3rd codon position parameters stabilized at different values in different runs and that the difference was most pronounced for the pinvar (I) and gamma (Г) parameters. When gamma alone was used to account for rate heterogeneity in this partition, the runs converged. The 3rd codon positions contained very few (2.1%) invariable sites, and there may have been insufficient data for estimating the pinvar parameter properly. All model parameters were unlinked across partitions and were allowed to evolve during the run starting from flat priors. All analyses were performed with four independent runs, each with three heated and one cold chain. Preliminary runs revealed poor mixing for the rate multiplier parameter under the default tuning parameter value of 500, and the value was therefore increased to 8000 (less bold proposals). The analyses were run for 100 million generations with sampling every 10 000 generations. Convergence was assessed by examining the average standard deviation of split frequencies between the four runs, the potential scale reduction factor for each model parameter and the mixing behaviour of the model parameters. The average standard deviation of split frequencies was calculated using a custom C++ program, SplitFreqs, which is available from the first author upon request. Model parameters and likelihood values were inspected in Tracer (Rambaut & Drummond 2007). The posterior tree distribution was summarized in a majority-rule consensus tree after discarding the first 25% of the samples as burn-in. The analyses were run at the Bioportal computer facility (http://www.bioportal.uio.no) at the University of Oslo, Norway.

## Results

### Sequence alignment

The concatenated sequence alignment of 180 samples included 3786 positions after trimming. Complete sequence information was obtained for 150 samples, while 30 lacked sequence data for one of the three target regions (Table 1). Because of alignment ambiguity, 284 positions were excluded from all phylogenetic analyses. Table S3 lists the number of parsimony informative, uninformative, invariant and excluded sites for each target gene. The alignment and partition definitions are included as a nexus file in the Supporting information (Data S1).

### Phylogenetic analyses

**Figure 1:**
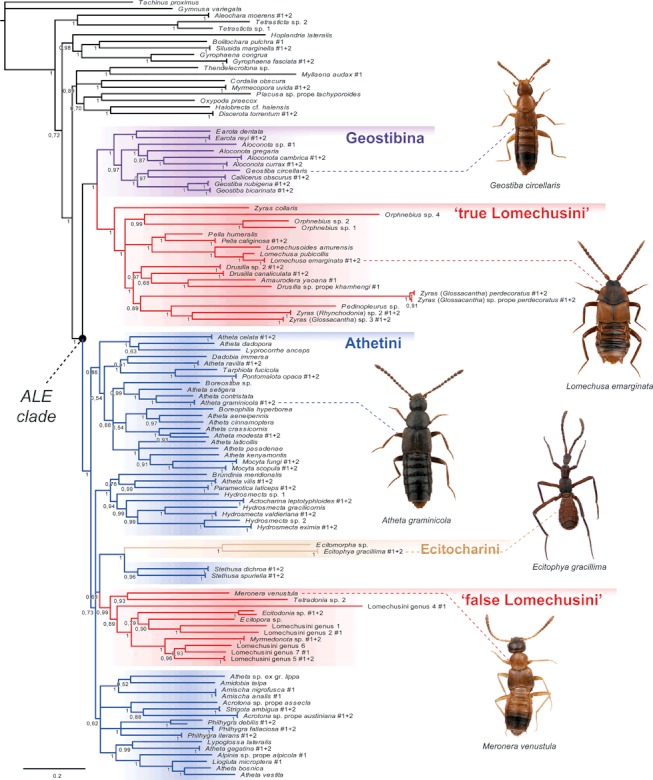
Majority-rule consensus tree from the Bayesian analysis with incomplete sequences excluded. Posterior probabilities are indicated under the branches. The labels of conspecific specimens have been combined to save space.

The majority-rule consensus trees from the Bayesian analyses are shown in Figs 1 (complete sequences only) and 2 (incomplete sequences included). The trees with the highest likelihood score from the ML analyses are shown in Figs S1 (complete sequences only) and S2 (incomplete sequences included). In both ML analyses, the highest-scoring trees were found by using bootstrap trees rather than randomized parsimony trees as the starting points for ML optimization.

**Figure 2:**
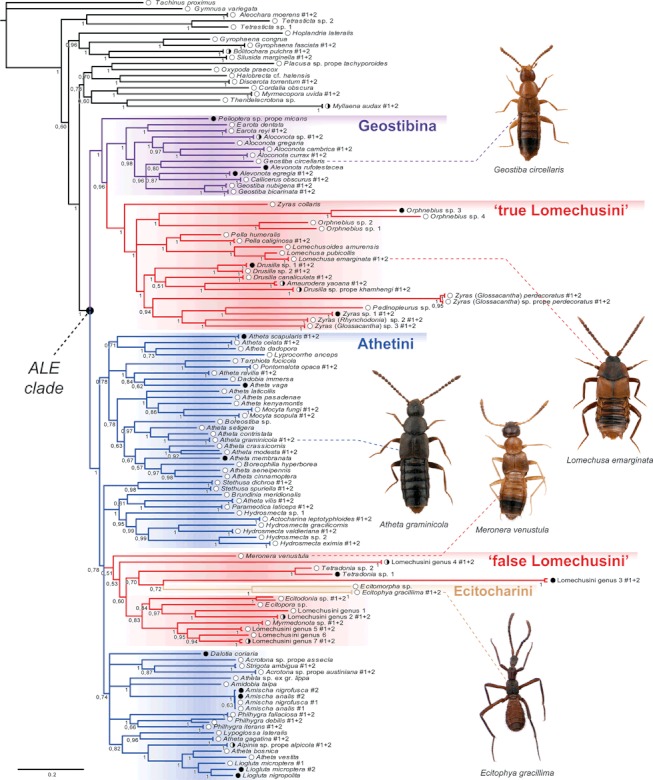
Majority-rule consensus tree from the Bayesian analysis with incomplete sequences included. Posterior probabilities are indicated under the branches. The labels of conspecific specimens have been combined to save space, except where the specimens did not group together. Complete sequences are indicated with open circles, incomplete with solid circles. Half-solid circles indicate pairs of conspecific specimens with one having incomplete sequence.

In both Bayesian analyses, the average standard deviation of split frequencies stabilized between 0.01 and 0.05 after about 20 million generations, indicating that stationarity was reached after about 5 million generations. The final standard deviation values after 100 million generations were 0.011 when incomplete sequences were excluded and 0.032 when they were included. The potential scale reduction factor approached 1 for all model parameters, never exceeding 1.009. Most model parameters showed good mixing, but the substitution rates and base frequencies for unpaired mitochondrial sites showed bimodal sampling when incomplete sequences were excluded. The chains swapped frequently between the two distinct optima, and both were thoroughly sampled during the run. The rate multiplier parameters showed alternating periods of good and poor mixing when the proposals tuning parameter value was adjusted to 8000, in contrast to uniformly poor mixing under the default value of 500.

The Bayesian and ML analyses produced largely congruent phylogenies (Figs 1, 2, S1 and S2) with very similar relative branch lengths, although the total inferred tree length was somewhat higher (up to 23%) under Bayesian inference. Many nodes were recovered with high statistical support both under ML and Bayesian inference. All trees contained some exceptionally long branches, particularly among the Lomechusini.

The ALE clade was recovered with high statistical support in all analyses (Bayesian posterior probability (PP) = 1.00, ML bootstrap support (BS) = 87%). When incomplete sequences were excluded (Figs 1 and S1), the Geostibina clade, represented by *Aloconota*, *Callicerus, Earota* and *Geostiba*, formed a sister group to the ‘true Lomechusini’ clade, which included *Amaurodera*, *Drusilla*, *Lomechusa*, *Lomechusoides*, *Orphnebius*, *Pedinopleurus*, *Pella* and *Zyras*. The Geostibina clade, the ‘true Lomechusini’ clade and the sister group relationship between them all received high statistical support (PP = 1.00, BS > 95%). In turn, the (Geostibina, ‘true Lomechusini’) clade formed a sister group to the main Athetini clade (PP = 1.00, BS = 83%), which included Ecitocharini and the ‘false Lomechusini’. The ‘false Lomechusini’ clade, consisting of *Ecitodonia*, *Ecitopora*, *Meronera*, *Myrmedonota*, *Tetradonia* and several not yet identified Neotropical taxa, was well supported in the Bayesian analysis (PP = 0.99) but not in the ML analysis (BS < 50%). The Ecitocharini, represented by two genera, were recovered as monophyletic (PP = 1.00, BS = 100%) and had *Stethusa* as its sister group (PP = 1.00, BS = 93%).

The inclusion of 30 incomplete sequences had little impact on the overall tree topology (Figs 2 and S2). Of the well-supported relationships, only the (*Stethusa*, Ecitocharini) clade was not recovered when incomplete sequences were included; *Stethusa* and Ecitocharini instead formed separate clades with poorly supported or unresolved relationships to other athetine clades. Also, the ‘false Lomechusini’ were not recovered as monophyletic when incomplete sequences were included.

All five genera included in the Geostibina clade have sensillum *a* of the epipharynx reduced. Among the other genera included in our study, only *Pelioptera* shares this character state. When examining the microscope slides of the entomological collection of the Natural History Museum, Oslo, as well as published illustrations of athetine genera, we identified additional genera (not included in our phylogenies) that have sensillum *a* of the epipharynx reduced. Table 2 lists taxa in which the presence of reduced sensillum *a* has been confirmed. In total, 13 genera were confirmed to share this character state. For nine of these, the type species was examined. Six other athetine genera that resemble *Geostiba* in having the ligula fully bilobed were found to have sensillum *a* fully developed (i.e. much longer than wide): *Boreophilia* (slide preparation examined), *Liogluta* (see Yosii & Sawada 1976: fig. 40B), *Madeirostiba* (see Assing & Wunderle 1995: fig. 5), *Ousipalia* (slide preparation examined), *Saphocallus* (see Assing 2001: fig. 16a), *Schistoglossa* (slide preparation examined) and *Tomoglossa* (see Sawada 1977: fig. 9c).

**Table 2:** Genera and species confirmed to belong to the tribe Geostibini based on the presence of reduced sensillum *a* of the epipharynx. Type species are indicated in bold

Genus	Species	Examined material/publications
*Alevonota* Gravenhorst, 1802	*A. gracilenta* (Erichson, 1839)	ZMUN slide collection
*Aloconota* Thomson, 1858	*A. brunneipes* (Casey, 1906)	ZMUN slide collection
*A. bulbosa* Sawada, 1989	Sawada (1989a): fig. 12b
*A. cuspidata* (Sawada, 1971)	Sawada (1971): fig. 2c (as *Tomoglossa*)
***A. gregaria*** (**Erichson, 1839**)	Yosii & Sawada (1976): fig. 43b
*A. insecta* (Thomson, 1856)	Yosii & Sawada (1976): fig. 44b
*A. languida* (Erichson, 1839)	Sawada (1984): fig. 3b (as *Disopora*)
*A. pfefferi* (Roubal, 1929)	ZMUN slide collection
*A. punctifoveata* (Sawada, 1970)	Sawada (1970): fig. 8c (as *Tomoglossa*)
*A. sulcifrons* (Stephens, 1832)	ZMUN slide collection
*Callicerus* Gravenhorst, 1802	***C. obscurus* Gravenhorst, 1802**	Yosii & Sawada (1976): fig. 46b Assing (2001): fig. 2b
*C. rigidicornis* (Erichson, 1839)	Assing (2001): fig. 14a
*Chinecallicerus* Assing, 2004	*C. laevigatus* Assing, 2006	Assing (2006): fig. 4
*Earota* Mulsant & Rey, 1874	*E. dentata* (Bernhauer, 1906)	Gusarov (2002b): fig. 2
*Enalodroma* Thomson, 1859	***E. hepatica*** (**Erichson, 1839**)	Sawada (1984): fig. 4b (as *Aloconota*)
*Geostiba* Thomson, 1858	*G. alticola* Lohse & Smetana, 1988	ZMUN slide collection
*G. appalachigena* Gusarov, 2002	ZMUN slide collection
*G. balsamensis* Pace, 1997	ZMUN slide collection
*G. bicarinata* Lohse & Smetana, 1988	ZMUN slide collection
*G. carteriensis* Pace, 1997	Gusarov (2002a): fig. 4
***G. circellaris*** (**Gravenhorst, 1806**)	Yosii & Sawada (1976): fig. 47b Gusarov (2002a): fig. 2
*G. crepusculigena* Gusarov, 2002	ZMUN slide collection
*G. daisetsuana* Sawada, 1989	Sawada (1989b): fig. 3b
*G. flava* (Kraatz, 1856)	ZMUN slide collection
*G. graveyardensis* Pace, 1997	ZMUN slide collection
*G. infirma* (Weise, 1878)	ZMUN slide collection
*G. nebuligena* Gusarov, 2002	ZMUN slide collection
*G. pluvigena* Gusarov, 2002	ZMUN slide collection
*G. sakhalinensis* Pace, 1997	ZMUN slide collection
*G. winkleri* (Bernhauer, 1915)	ZMUN slide collection
*Homoiocalea* Bernhauer, 1943	*H. toroenensis* (Bernhauer, 1943)	Sawada (1984): fig. 1b (as *Callicerus*)
*Micrearota* Casey, 1910	*M. prolongata* (Casey, 1910)	ZMUN slide collection
*Pelioptera* Kraatz, 1857	*P. acuticollis* (Kraatz, 1859)	Sawada (1982): fig. 10b
*P. babai* Sawada, 1989	Sawada (1989a): fig. 13b
*P. exasperata* (Kraatz, 1859)	Sawada (1982): fig. 11b
*P. flavonitescens* (Bernhauer, 1938)	Sawada (1977): fig. 17k (as *Geostiba*)
*P. luzonica* (Bernhauer, 1916)	Sawada (1980): fig. 16b
*P. micans* (Kraatz, 1857)	Sawada (1980): fig. 9b
*P. monticola* Cameron, 1933	Sawada (1980): fig. 15b
*P. nilgiriensis* (Fauvel, 1904)	Sawada (1980): fig. 11b
*P. opaca* (Kraatz, 1857)	Sawada (1980): fig. 10b
*P. ocyamensis* (Bernhauer, 1914)	Sawada (1977): fig. 17b (as *Geostiba*)
*P. peguana* (Bernhauer, 1915)	Sawada (1980): fig. 14b
*P. purpurascens* (Cameron, 1920)	Sawada (1987): fig. 5b
*P. testaceipennis* (Motschulsky, 1858)	Sawada (1977): fig. 18b (as *Geostiba luchuensis* (Cameron, 1933) Sawada (1980): fig. 13b
*P. vacillator* (Cameron, 1933)	Sawada (1977): fig. 19b (as *Geostiba*)
*P. xylophila* (Cameron, 1920)	Sawada (1980): fig. 12b
*Pseudosemiris* Machulka, 1935	***P****.* ***kaufmanni*** (**Eppelsheim, 1887**)	Assing (2001): fig. 19a
*Seeversiella* Ashe, 1986	***S****.* ***globicollis*** (**Bernhauer, 1907**)	Gusarov (2003): fig. 2
*Tropimenelytron* Pace, 1983	*T. americanum* Gusarov, 2002	ZMUN slide collection
***T****.* ***tuberiventre*** (**Eppelsheim *in* Leder, 1879**)	Gusarov (2002c): fig. 2
*T. unicum* (Bernhauer, 1907)	Sawada (1977): fig. 15b (as *Aloconota*)

## Discussion

The molecular phylogeny of the Athetini–Lomechusini–Ecitocharini (ALE) clade of aleocharine rove beetles presented in this study is in line with the main finding of Elven *et al.* (2010). The monophyly of the ALE clade as well as the three major clades within it, the Geostibina, the ‘true Lomechusini’, and the main Athetini clade, was confirmed with high statistical support. All non-geostibine Athetini except *Discerota torrentum* were recovered as part of the main Athetini clade, which also included Ecitocharini and the ‘false Lomechusini’. Of the five initial hypotheses to be tested in this study, none was rejected. There was a strong statistical support for all hypothesized relationships except the monophyly of the ‘false Lomechusini’ (hypothesis 4), for which there was only moderate support. The five hypotheses are reviewed below.

H1: Geostibina are a sister clade to the ‘true Lomechusini’.

The Geostibina clade and the ‘true Lomechusini’ clade were both strongly supported, and the sister group relationship between them strongly corroborated.

Within the Geostibina clade, the monophyly of *Earota* (two species) and *Aloconota* (four species) was confirmed, but the monophyly of *Geostiba* (three species) and *Alevonota* (two species) was not. The inclusion of *Callicerus* in the Geostibina clade creates a nomenclatural problem. The rarely used family group name Callicerina Jakobson, 1908 has priority over the currently more widely used Geostibina Seevers, 1978; but the first is also a junior homonym of Callicerini Rondani, 1845, which is in use for a tribe of hoverflies (Diptera: Syrphidae). To solve the issue, an application proposing to suppress the name Callicerina Jakobson, 1908 and to keep the more widely used Geostibina Seevers, 1978 has been submitted to the International Commission on Zoological Nomenclature (Gusarov 2011). Pending the Commission’s ruling, we maintain current usage and treat the name Geostibina as valid.

The ‘true Lomechusini’ clade included *Drusilla*, *Lomechusa*, *Pella* and five other genera represented here by species from the Old World. Within this clade, the genera *Lomechusa* (two species), *Orphnebius* (four species) and *Pella* (two species) were recovered as monophyletic with high support. The genus *Drusilla* (four species) was recovered as paraphyletic with respect to the morphologically distinct Oriental genus *Amaurodera* (one species). As currently accepted, *Drusilla* is distributed worldwide and includes almost 200 species (Hlaváč *et al.* 2011). Within *Drusilla*, there is a fairly distinct group of Palaearctic species related to the type species of the genus, *Drusilla canaliculata* (revised by Assing 2005). However, *Drusilla* in the broad sense can only be defined by the lack of unusual characters (e.g. the distinct shape of pronotum in *Amaurodera*). A worldwide revision of *Drusilla* is needed to divide the genus into diagnosable monophyletic genera. The genus *Zyras* (represented in our analyses by six species) was recovered as polyphyletic. The nominotypical subgenus (represented by *Z. collaris*) was recovered as the sister group to all other ‘true Lomechusini’. Two closely related species of *Zyras* (*Glossacantha*) from Thailand were recovered in a separate clade with a very long branch. A third clade included yet another species of *Zyras* (*Glossacantha*), one species of *Zyras* (*Rhynchodonia*), one *Zyras* species not yet identified to subgenus and the morphologically very distinct genus *Pedinopleurus*. Accordingly, even the subgenus *Glossacantha* is not monophyletic. In the current classification, Zyras is divided into 54 subgenera and includes more than 800 species (Hlaváč *et al.* 2011). Some groups formerly treated as subgenera of *Zyras* have been raised to genus rank (e.g. *Pella*: Kistner 1972; Maruyama 2006) and new subgenera are being described regularly (e.g. Pace 1999). Like *Drusilla*, *Zyras* is a group in need of a worldwide revision. Our results confirm the opinion of Kistner (1972) and Maruyama (2006) that *Pella* is not related to *Zyras* and should be treated as a separate genus.

H2: Geostibina and the ‘true Lomechusini’ form a sister group to the main Athetini clade.

The main Athetini clade was recovered with strong support in a strongly supported sister group relationship with Geostibina and the ‘true Lomechusini‘. The basal nodes of the main Athetini clade were not resolved with good support.

H3: All athetine genera with sensillum *a* of the epipharynx reduced belong to Geostibina.

All five genera forming the Geostibina clade (*Alevonota*, *Aloconota*, *Callicerus*, *Earota* and *Geostiba*) have sensillum *a* of the epipharynx reduced. Among the other genera included in this study, only *Pelioptera* has this character state. In our analyses, *Pelioptera* formed a sister group to the (Geostibina, ‘true Lomechusini’) clade. The support for this placement was not strong, however, and is further weakened by the fact that the entire COI–COII region was missing from the *Pelioptera* sequence. Given that *Pelioptera* shares additional character states (see below) with the five genera of Geostibina, we hypothesize that *Pelioptera* is also a member of Geostibina and that the reduced sensillum *a* is a synapomorphy for this group. In addition to the six genera included in the molecular analyses, seven further athetine genera were confirmed to possess the reduced sensillum *a* of the epipharynx (Table 2). All 13 genera also share the shape of the ligula: broad at the base and divided into two separate lobes. We hypothesize that they all belong to Geostibina. Within Athetini, there are additional genera with a fully bilobed ligula. Two of these, *Liogluta* and *Boreophilia*, were included in our study and were not recovered as members of the Geostibina clade. Based on our examination, they also have a normally developed sensillum *a*. Five more genera with a bilobed ligula not included in this study (*Madeirostiba*, *Ousipalia*, *Saphocallus*, *Schistoglossa* and *Tomoglossa*) were also confirmed to have a normally developed sensillum *a*. Based on this evidence, we predict that they do not belong to the Geostibina clade.

H4: The ‘false Lomechusini’ are nested within the main Athetini clade.

The ‘false Lomechusini’ clade included *Meronera*, *Myrmedonota* and 10 other genera (11 species) from the New World. Seven of the included genera were unidentified. The clade was well supported only in the Bayesian analysis (Fig. 1), and when incomplete sequences were included, the ‘false Lomechusini’ were no longer recovered as monophyletic. The position of the ‘false Lomechusini’ within the main Athetini clade was not resolved. However, the ‘false Lomechusini’ are confirmed to belong to the main Athetini clade and thus not to be monophyletic with the ‘true Lomechusini’.

Ten of the included genera were collected in Ecuador and two in the USA. Of the five identified genera, two (*Ecitopora* and *Ecitodonia*) are exclusively Neotropical. Two genera (*Tetradonia* and *Meronera*) are distributed mostly in the Neotropical region, with a few species also in the Nearctic. The fifth genus, *Myrmedonota*, is known from both the Nearctic and the Oriental regions (but see below). The geographic distribution of the ‘false Lomechusini’ suggests that the clade may have originated in South America and dispersed into North America. More extensive taxon sampling in both Old and New World tropics is needed to test this hypothesis.

The placement of *Meronera* and *Myrmedonota* in the ‘false Lomechusini’ clade is in line with Elven *et al.* (2010). The genus *Myrmedonota* was represented by a species from the Eastern USA, but was originally described from Singapore (Cameron 1920) and is furthermore known from Malaysia, Indonesia and Papua New Guinea. It was only recently reported from North America by Maruyama *et al.* (2008), who provided a new diagnosis of the genus based on the type species *M. cingulata*, two new species from the Eastern United States and published descriptions of two species from New Guinea. The geographic distribution of *Myrmedonota* suggests that the Nearctic species may not be related to and congeneric with the Oriental. We do not propose to remove *Myrmedonota* from Lomechusini until additional species, in particular the type, have been examined in more detail.

H5: Ecitocharini form a monophyletic sister group to *Stethusa* within the main Athetini clade.

The Ecitocharini were recovered as a strongly supported monophyletic group within the main Athetini clade. When only complete sequences were used, Ecitocharini formed a well-supported sister group to the New World athetine genus *Stethusa* (Figs 1 and S1). However, when taxa with incomplete sequences were included in the analysis, Ecitocharini grouped (with weak support) with the longest branch of the ‘false Lomechusini’ formed by the unidentified genus 3 (Figs 2 and S2). This genus lacked sequence data for the COI–Leu2–COII region, and the 18S sequences of both genus 3 and the Ecitocharini were unusually divergent. We consider the weakly supported sister group relationship between Ecitocharini and genus 3 to be an artefact and treat the well-supported sister group relationship between Ecitocharini and *Stethusa* as phylogenetically correct.

The type genus of Ecitocharini, *Ecitochara*, was unfortunately not available for this study, which instead included the genera *Ecitophya* and *Ecitomorpha* as representatives of the tribe. However, the members of Ecitocharini share several derived morphological character states (Kistner & Jacobson 1990) and are furthermore connected by life style (i.e. association with army ants of the genus *Eciton*) and geographic distribution. It seems reasonable to assume that the tribe is monophyletic.

As the Ecitocharini are nested within the main Athetini clade, their inclusion in Athetini should be uncontroversial. Seevers (1965) rationale for erecting the tribe Ecitocharini was to ‘emphasize their evolutionary and ecological divergence from the Athetini’, but he believed that the former were derived from the latter, which is congruent with this study. We therefore place the name Ecitocharini Seevers, 1965 in synonymy with Athetini Casey, 1910. Ecitocharina may still be used as a valid name at the rank of subtribe, but lack of resolution at the base of the main Athetini clade does not currently allow us to divide the entire tribe into subtribes.

### Tribe-level classification of the ALE clade

The aim of this study was to resolve the phylogeny of the major lineages of the ALE clade in order to revise the classification of the tribes involved. With strong support for all three subclades within the ALE clade, the ‘true Lomechusini’, the subtribe Geostibina and the main Athetini clade, we can address the issue of classification. A revised tribe-level classification needs to meet the following criteria: (i) all formally recognized taxa should be monophyletic; (ii) the classification should reflect the three main subclades and be compatible with their phylogenetic relationships: [(Geostibina, Lomechusini) (the main Athetini clade including the ‘false Lomechusini’)]; (iii) the principle of priority should be satisfied; (iv) the recognized family group taxa should be diagnosable using morphological characters, preferably apomorphic states; and (v) the choice of classification should promote stability of scientific names (ICZN, 1999: Preamble). We here discuss three possible alternative classifications, all of which satisfy criteria 1–3 and all of which meet criterion 4 equally well by recognizing the three main subclades of the ALE clade in one way or another. The main difference between the three alternatives is in the ranks of some taxa. Therefore, we will focus on how the changes in classification will affect the stability of names (criterion 5).

**Alternative 1: raising the rank of Geostibina to tribe.** Three tribes are recognized in the ALE clade: Athetini, Geostibini and Lomechusini. Most of the genera and species currently in Athetini and Lomechusini stay in their respective tribes. The members of subtribe Geostibina (most of which belong to the genus *Geostiba*) are removed from Athetini by raising the rank of the subtribe to tribe. The genera of the ‘false Lomechusini’ are moved from Lomechusini to Athetini. Further subdivision of the three tribes into subtribes is still possible.

**Alternative 2: moving subtribe Geostibina to Lomechusini.** Two tribes are recognized in the ALE clade: Athetini and Lomechusini (including subtribe Geostibina). Most of the genera and species currently in Athetini and Lomechusini stay in their respective tribes. The subtribe Geostibina is moved from Athetini to Lomechusini, while the genera of ‘false Lomechusini’ are moved from Lomechusini to Athetini. Subdivision of the two tribes into subtribes is still possible.

**Alternative 3: expanding the Lomechusini to include all Athetini.** Only one tribe is recognized in the ALE clade: Lomechusini. This tribe is further subdivided into three subtribes: Athetina, Geostibina and Lomechusina. This solution is similar to some earlier classifications (e.g. by Bernhauer & Scheerpeltz 1926) where Athetini and Lomechusini were treated as subtribes of the tribe Myrmedoniini Thomson, 1867. The members of ‘false Lomechusini’ stay in the tribe Lomechusini, but are moved to the subtribe Athetina. If this solution is implemented, hundreds of genera and thousands of species currently in Athetini will need to be moved to Lomechusini, and the largest aleocharine tribe will be abandoned. This solution does not promote stability of names. Furthermore, as the ranks of what are currently treated as tribes Athetini and Lomechusini are lowered to subtribes, it becomes impossible to recognize the taxa currently treated as subtribes of Athetini and Lomechusini, except by inserting a rank of infratribe.

The main difference between the three alternatives is in how the stability of classification is promoted. Alternative 1 seems to achieve that goal best and at the same time allows further subdivision of all three tribes into subtribes. We favour this alternative and, consequently, raise the rank of the subtribe Geostibina to tribe and move the genera of ‘false Lomechusini’ to Athetini.

## The redefined tribes Athetini, Lomechusini and Geostibini

Most of the Athetini can be diagnosed by a combination of the following characters: sensillum *a* of the epipharynx fully developed, galea of moderate length, mesocoxae narrowly or moderately separated, and mesoventral process not broad. Most of the Lomechusini can be diagnosed by a combination of the following characters: sensillum *a* of the epipharynx fully developed, galea significantly elongate, mesocoxae broadly separated, and mesoventral process broad and short. Both in Athetini and Lomechusini, there are exceptions that do not fit the above diagnoses, and a detailed morphological study will be needed to improve the diagnoses of the two tribes.

The main diagnostic character and putative synapomorphy for the tribe Geostibini is sensillum *a* of the epipharynx reduced (e.g. as in Yosii & Sawada 1976: fig. 47B). Geostibini share with Athetini and Lomechusini the tarsal formula 4-5-5 and the presence of the athetine bridge of the aedeagus. Like most Athetini, but unlike Lomechusini, Geostibini have the galea moderately long, mesocoxae narrowly or moderately separated, and mesoventral process not broad. Like Lomechusini and some Athetini, Geostibini have the ligula broad at the base and divided into two separate lobes. Table 2 lists all genera in which reduction in sensillum *a* of the epipharynx has been confirmed. We place all these genera in the tribe Geostibini. There is no doubt that additional genera will need to be transferred from Athetini to Geostibini. Unfortunately, the main diagnostic character of Geostibini, sensillum *a* of the epipharynx, is rarely described or illustrated in published papers. Thus, for most athetine genera, direct examination of slide mounted specimens will be needed to assess their tribal placement.

## *Genus* Discerota

The athetine genus *Discerota* (represented by *D. torrentum*) formed a well-supported clade with *Halobrecta* outside the ALE clade. In Elven *et al.* (2010), *Halobrecta* was removed from Athetini and tentatively placed in Oxypodini. Like *Halobrecta*, *Discerota* lacks the athetine bridge of the aedeagus and has both male and female genitalia similar to some Oxypodini. For these reasons, we move *Discerota* from Athetini and place it tentatively in Oxypodini as well.

## The riparian clade of Athetini

Compared to Elven *et al.* (2010), we expanded the taxon sampling of Athetini by adding several species associated with riparian habitats. In addition to providing a more rigorous test of the monophyly of the main Athetini clade, this allowed us to test whether the riparian athetines are related to each other or if different lineages have colonized the riparian habitats independently.

Remarkably, many riparian taxa were recovered as members of a strongly supported monophyletic group. This riparian clade included *Actocharina*, *Atheta* (*Dralica*) *vilis*, *Brundinia*, *Parameotica* and *Hydrosmecta* (represented by five species).

The monotypic genus *Actocharina*, originally a subgenus of *Atheta,* is distributed in Austria and northern Italy where it inhabits sandy river banks in the *Kalkalpen*. The beetles are minuscule, only up to 1.4 mm long, have reduced eyes, wings and pigmentation and presumably move in the interstitial space between sand particles like many *Hydrosmecta*. When describing *Atheta* (*Actocharina*) *leptotyphloides*, Bernhauer (1907) mentioned its close relationship to two small species of *Hydrosmecta*, *H. subtilissima* and *H. tenuissima*. With respect to external morphology, *Actocharina* is indeed similar to the smallest species of *Hydrosmecta*, for example, *H. delicatula* or *H. tenuissima* (cf. figs 162:1 and 164:13, 17 in Benick & Lohse 1974). The main difference between the two genera is *Actocharina* being more derived in characters related to cryptic interstitial life style. In our analyses, *Actocharina* formed a well-supported clade with four of the five included species of *Hydrosmecta*, and the fifth *Hydrosmecta* species was sister to this clade. We, therefore, consider *Actocharina* a morphologically derived member of the genus *Hydrosmecta* and place the name *Actocharina* Bernhauer, 1907 in synonymy with *Hydrosmecta* Thomson, 1858.

## Conclusions

Elven *et al.* (2010) demonstrated that the tribes Athetini and Lomechusini are not monophyletic and that the tribe Ecitocharini may belong to Athetini. In this study, we thoroughly assessed the basal relationships among the three tribes to propose a phylogenetically robust tribe-level classification. The athetine subtribe Geostibina was shown to be a sister group to the ‘true Lomechusini’. Five athetine genera are included in this clade, and eight more can be referred to it based on morphology. Geostibina and the ‘true Lomechusini’ together form a sister group to the main Athetini clade, which comprises all non-geostibine athetines in addition to the tribe Ecitocharini and the ‘false Lomechusini’. The resolution within the main Athetini clade was poor, but support for the clade itself was strong. The monophyly of the ‘false Lomechusini’ was not strongly supported; nevertheless, there is no doubt about their inclusion in Athetini and their separation from the ‘true Lomechusini’. We propose raising the subtribe Geostibina to the rank of tribe. Doing so will best promote stability of nomenclature, while complying with the criterion of monophyly. We furthermore propose including the ‘false Lomechusini’ and the Ecitocharini in Athetini.

It is likely that future revisional work on Athetini and Lomechusini and the inclusion of further genera in large-scale phylogenetic analyses will further change the definition of these tribes. The ‘false Lomechusini’ in particular raise many further questions, such as whether the group is actually monophyletic, which other New World genera belong to it, whether the group is also represented outside the New World, and which are its closest relatives within Athetini. Further studies are also needed on phylogeny of the ‘true Lomechusini’. The molecular markers used in this study may prove suitable for resolving relationships within *Zyras* and *Drusilla*, the two most challenging genera within the Lomechusini.

### Proposed changes in classification

The following changes in classification of Aleocharinae are proposed (see Table 3 for details): (i) Geostibina, formerly a subtribe of Athetini, is raised to tribe rank as Geostibini Seevers, 1978, *stat. nov*. Thirteen genera are moved from Athetini to Geostibini. (ii) Three genera are moved from Lomechusini to Athetini. (iii) The family group name Ecitocharini Seevers, 1965 is placed in synonymy with the name Athetini Casey, 1910. All 10 genera formerly treated as members of the tribe Ecitocharini are moved to Athetini. (iv) The genus *Discerota* Mulsant & Rey, 1874 is removed from Athetini and tentatively included in Oxypodini. (v) The genus name *Actocharina* Bernhauer, 1907 is placed in synonymy with *Hydrosmecta* Thomson, 1858. The new combination *Hydrosmecta leptotyphloides* (Bernhauer, 1907) is established for the species originally described as *Atheta* (*Actocharina*) *leptotyphloides* Bernhauer, 1907.

**Table 3:** Proposed changes in classification of Aleocharinae

Name	Previous status/placement	New status/placement
Geostibina Seevers, 1978	Valid subtribe of Athetini	Tribe Geostibini Seevers, 1978, *stat. nov*.
*Alevonota* Thomson, 1858	Athetini	Geostibini
*Aloconota* Thomson, 1858	Athetini	Geostibini
*Callicerus* Gravenhorst, 1802	Athetini	Geostibini
*Chinecallicerus* Assing, 2004	Athetini	Geostibini
*Earota* Mulsant & Rey, 1874	Athetini	Geostibini
*Enalodroma* Thomson, 1859	Athetini	Geostibini
*Geostiba* Thomson, 1858	Athetini	Geostibini
*Homoiocalea* Bernhauer, 1943	Athetini	Geostibini
*Micrearota* Casey, 1910	Athetini	Geostibini
*Pelioptera* Kraatz, 1857	Athetini	Geostibini
*Pseudosemiris* Machulka, 1835	Athetini	Geostibini
*Seeversiella* Ashe, 1986	Athetini	Geostibini
*Tropimenelytron* Pace, 1983	Athetini	Geostibini
*Ecitodonia* Seevers, 1965	Lomechusini	Athetini
*Ecitopora* Wasmann, 1887	Lomechusini	Athetini
*Tetradonia* Wasmann, 1894	Lomechusini	Athetini
Ecitocharini Seevers, 1965	Valid tribe	New synonym of Athetini Casey, 1910
*Campbellia* Kistner & Jacobson, 1990	Ecitocharini	Athetini
*Ecitochara* Wasmann, 1887	Ecitocharini	Athetini
*Ecitodaemon* Reichensperger, 1939	Ecitocharini	Athetini
*Ecitomorpha* Wasmann, 1889	Ecitocharini	Athetini
*Ecitophya* Wasmann, 1900	Ecitocharini	Athetini
*Ecitoschneirla* Kistner & Jacobson, 1990	Ecitocharini	Athetini
*Ecitosymbia* Bruch, 1923	Ecitocharini	Athetini
*Ecitoxenia* Wasmann, 1900	Ecitocharini	Athetini
*Retteneciton* Kistner & Jacobson, 1990	Ecitocharini	Athetini
*Seeverseciton* Kistner & Jacobson, 1990	Ecitocharini	Athetini
*Discerota* Mulsant & Rey, 1874	Athetini	Tentatively Oxypodini
*Actocharina* Bernhauer, 1907	Valid genus	New synonym of *Hydrosmecta* Thomson, 1858
*Actocharina leptotyphloides* (Bernhauer, 1907)	*Actocharina*	*Hydrosmecta leptotyphloides* (Bernhauer, 1907), new combination

## Supporting Information

Fig. S1 Best tree from the maximum likelihood analysis with incomplete sequences excluded. Bootstrap values ≥ 50% are indicated under the branches. The labels of conspecific specimens have been combined to save space.

Fig. S2 Best tree from the maximum likelihoodanalysis with incomplete sequences included. Bootstrap values ≥ 50% are indicated under the branches. The labels of conspecific specimens have been combined to save space, except where the specimens did not group together. Complete sequences are indicated with open circles, incomplete with solid circles. Half-solid circles indicate pairs of conspecific specimens with one having incomplete sequence.

Table S1 Label information for the specimens included in this study. The specimens marked with asterisk (*) are on loan from the Natural History Museum of Denmark (ZMUC) and will be divided between ZMUC and the Natural History Museum, University of Oslo (ZMUN) upon completion of the project. The remaining specimens are deposited at ZMUN.

Table S2 Primers used for amplification andsequencing. Alternative primers used to amplify certain difficultsamples are indicated with ‘alt’. Primers usedexclusively as internal sequencing primers are indicated with‘int’.

Table S3 Alignment statistics on the molecular markers used in the study. The four genes COI, COII, 16S and 18S account for 94% of the total alignment, or 3568 sites.

Data S1 ALE_alignment.nex. Sequence alignment in nexus format. Concatenated alignment of two mitochondrial and one nuclear region, covering 3568 bases for 180 samples.
