# Supplementary material for: Molecular phylogeny of the Athetini–Lomechusini–Ecitocharini clade of aleocharine rove beetles (Insecta)
Source: Zool Scr. 2012 Jun 20;41(6):617–36. doi: 10.1111/j.1463-6409.2012.00553.x (PMC3532658; doi:10.1111/j.1463-6409.2012.00553.x)
Supplement: Fig. S2 — Best tree from the maximum likelihoodanalysis with incomplete sequences included. Bootstrap values ≥ 50% are indicated under the branches. The labels of conspecific specimens have been combined to save space, except where the specimens did not group together. Complete sequences are indicated with open circles, incomplete with solid circles. Half-solid circles indicate pairs of conspecific specimens with one having incomplete sequence. [file zsc0041-0617-SD2.pdf]

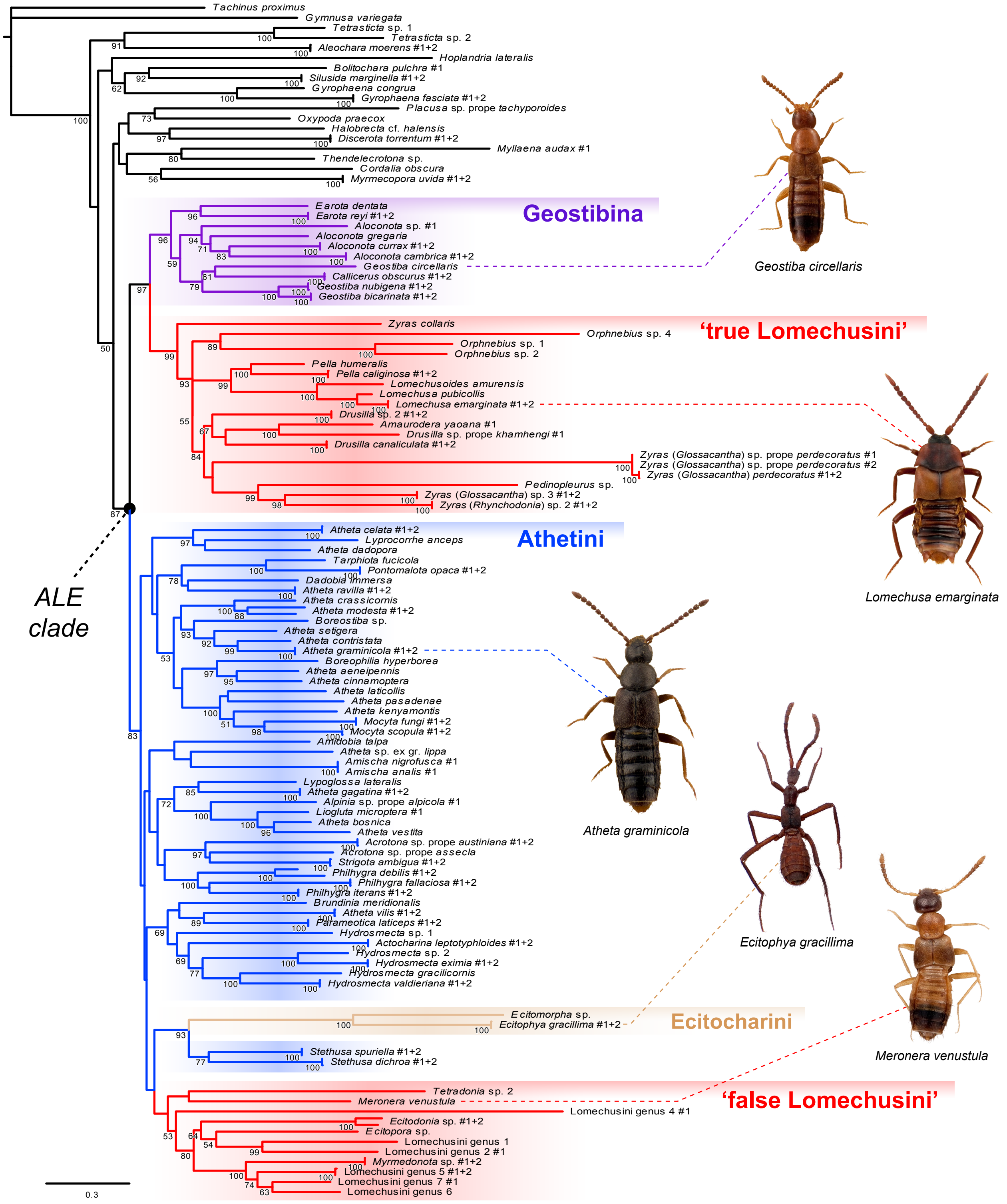

Supplementary Figure S1 Best tree from the Maximum Likelihood analysis with incomplete sequences excluded. Bootstrap values ≥ 50% are indicated under the branches. The labels of conspecific specimens have been combined to save space.
