# Supplementary material for: Molecular phylogeny of the Athetini–Lomechusini–Ecitocharini clade of aleocharine rove beetles (Insecta)
Source: Zool Scr. 2012 Jun 20;41(6):617–36. doi: 10.1111/j.1463-6409.2012.00553.x (PMC3532658; doi:10.1111/j.1463-6409.2012.00553.x)
Supplement: Table S1 — Label information for the specimens included in this study. The specimens marked with asterisk (*) are on loan from the Natural History Museum of Denmark (ZMUC) and will be divided between ZMUC and the Natural History Museum, University of Oslo (ZMUN) upon completion of the project. The remaining specimens are deposited at ZMUN. [file zsc0041-0617-SD3.pdf]

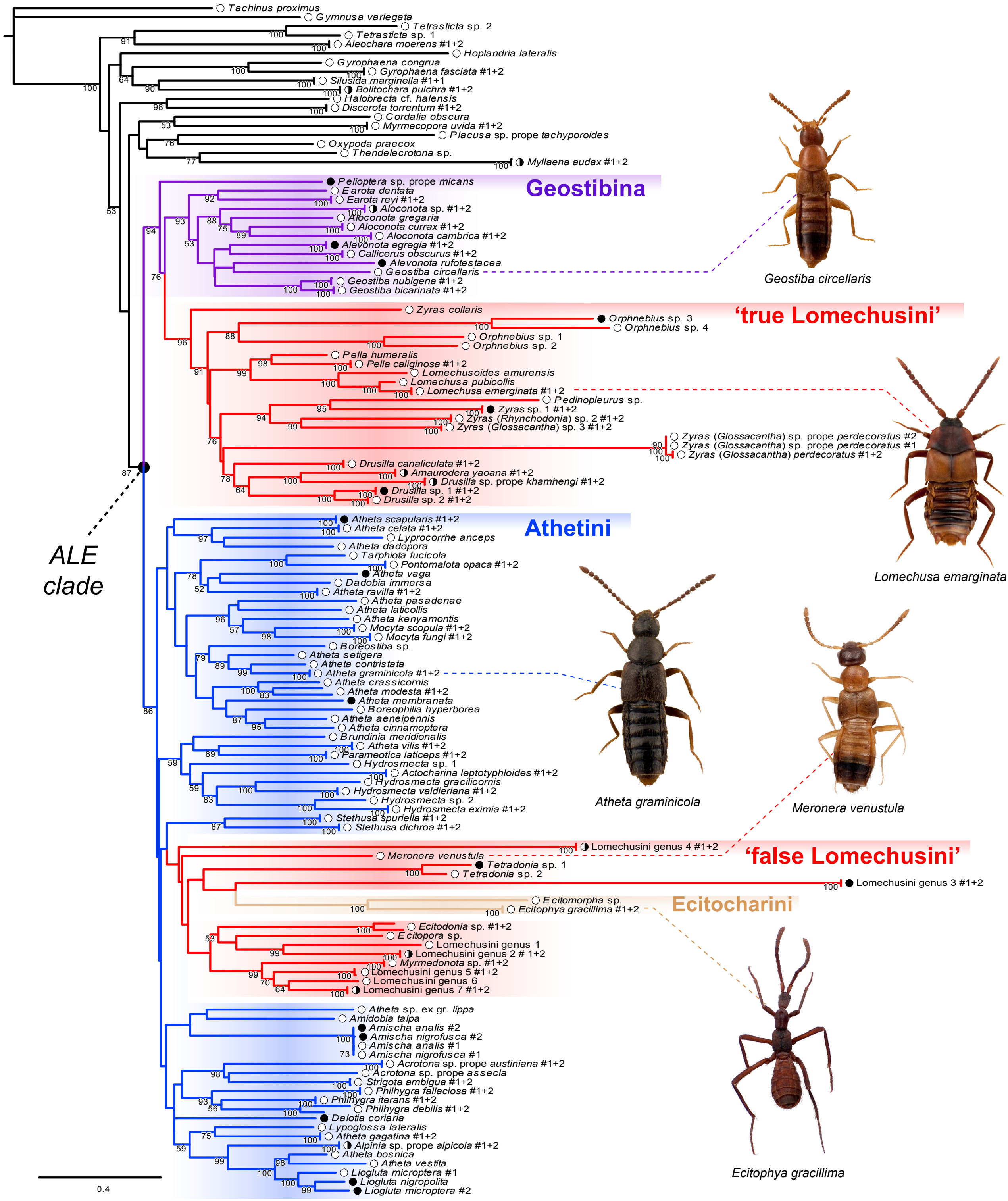

Supplementary Figure S2 Best tree from the Maximum Likelihood analysis with incomplete sequences included. Bootstrap values ≥ 50% are indicated under the branches. The labels of conspecific specimens have been combined to save space, except where the specimens did not group together. Complete sequences are indicated with open circles, incomplete with solid circles. Half-solid circles indicate pairs of conspecific specimens with one having incomplete sequence.
