# Supplementary material for: Molecular phylogeny of the Athetini–Lomechusini–Ecitocharini clade of aleocharine rove beetles (Insecta)
Source: Zool Scr. 2012 Jun 20;41(6):617–36. doi: 10.1111/j.1463-6409.2012.00553.x (PMC3532658; doi:10.1111/j.1463-6409.2012.00553.x)
Supplement: Table S2 — Primers used for amplification andsequencing. Alternative primers used to amplify certain difficultsamples are indicated with ‘alt’. Primers usedexclusively as internal sequencing primers are indicated with‘int’. [file zsc0041-0617-SD4.pdf]

Supplementary Table S1 Label information for the specimens included in this study. The specimens marked with asterisk (*) are on loan from the Natural History Museum of Denmark (ZMUC) and will be divided between ZMUC and the Natural History Museum, University of Oslo (ZMUN) upon completion of the project. The remaining specimens are deposited at ZMUN.

ZMUN barcode	Species	Label information
10002544	*Acrotona* sp. prope *assecla* (Casey, 1910)	U.S.A., Tennessee, Sevier Co., 9.5 km NEE Gatlinburg, Great Smoky Mountains National Park, Greenbrier, nr. entrance, 35°44.265'N 83°24.976'W ±6m, h=455m, in hay [2447] [Garmin eTrex; WGS84], 30.vi.2006, Leg. V.I.Gusarov
10002543	*Acrotona* sp. prope *austiniana* (Casey, 1910)	U.S.A., Tennessee, Sevier Co., 11.5 km E Gatlinburg, Great Smoky Mountains National Park, Greenbrier, pavilion, 35°42.219'N 83°23.165'W ±9m, h=575m, light trap [2419] [Garmin eTrex; WGS84], 24.vi.2006, Leg. V.I.Gusarov
10002547	*Acrotona* sp. prope *austiniana* (Casey, 1910)	U.S.A., Tennessee, Sevier Co., 11.5 km E Gatlinburg, Great Smoky Mountains National Park, Greenbrier, pavilion, 35°42.219'N 83°23.165'W ±9m, h=575m, light trap [2419] [Garmin eTrex; WGS84], 24.vi.2006, Leg. V.I.Gusarov
10002656	*Actocharina leptotyphloides* (Bernhauer, 1907)	Austria, Tirol, Bezirk Reutte, W of Stanzach, nr. Hwy. 198 Lech River (left bank opposite Stanzach), 47°22.938'N 10°33.153'E ±12m, h=942m, flooding river banks [3348] [Garmin 60CSx; WGS84], 10.v.2008, Leg. V.I.Gusarov & H.Elven
10002657	*Actocharina leptotyphloides* (Bernhauer, 1907)	Austria, Tirol, Bezirk Reutte, W of Stanzach, nr. Hwy. 198 Lech River (left bank opposite Stanzach), 47°22.938'N 10°33.153'E ±12m, h=942m, flooding river banks [3348] [Garmin 60CSx; WGS84], 10.v.2008, Leg. V.I.Gusarov & H.Elven
10002579	*Aleochara moerens* Gyllenhal, 1827	Norway, Sør-Trøndelag, Røros kommune, 31 km SE Røros, 3 km NEE Langen, Rundtjønna Lake, NW bank, 62°26.784'N 11°54.583'E ±8m, h=659m, in fungi [2173] [Garmin eTrex; WGS84], 15.viii.2005, Leg. V.I.Gusarov
10002570	*Aleochara moerens* Gyllenhal, 1827	Norway, Sør-Trøndelag, Røros kommune, 31 km SE Røros, 3 km NEE Langen, Rundtjønna Lake, NW bank, 62°26.784'N 11°54.583'E ±8m, h=659m, in fungi [2173] [Garmin eTrex; WGS84], 15.viii.2005, Leg. V.I.Gusarov
10030889	*Alevonota egregia* (Rye, 1876)	France, Pyrénées-Orientales, forêt de Rabouillet-Boucheville, h=800–1100m, filet de voiture, 6.v.2008, Leg. M.Tronquet
10030891	*Alevonota egregia* (Rye, 1876)	France, Pyrénées-Orientales, forêt de Rabouillet-Boucheville, h=800–1100m, filet de voiture, 6.v.2008, Leg. M.Tronquet
10030810	*Alevonota rufotestacea* (Kraatz, 1856)	France, Pyrénées-Orientales, forêt de Rabouillet-Boucheville, h=800–1100m, filet de voiture, 6.v.2008, Leg. M.Tronquet

10030823	*Aloconota cambrica* (Wollaston, 1855)	Austria, Tirol, Bezirk Reutte, 5 km SW Reutte, nr. Hwy. 198 Lech River (left bank), 47°27.049'N 10°39.777'E ±4m, h=870m, near river, bare ground [3345] [Garmin 60CSx; WGS84], 10.v.2008, Leg. V.I.Gusarov
10029295	*Aloconota cambrica* (Wollaston, 1855)	Austria, Oberösterreich, Bezirk Kirchdorf an der Krems, 1 km N Molln, Gstadt, Krumme Steyrling, 47°53.676'N 14°15.351'E ±6m, h=383m, flooding river banks, stones, gravel, sand [3312] [Garmin 60CSx; WGS84], 6.v.2008, Leg. V.I.Gusarov & H.Elven
10029300	*Aloconota currax* (Kraatz, 1856)	Austria, Oberösterreich, Bezirk Kirchdorf an der Krems, 1 km N Molln, Gstadt, Krumme Steyrling, 47°53.676'N 14°15.351'E ±6m, h=383m, flooding river banks, stones, gravel, sand [3312] [Garmin 60CSx; WGS84], 6.v.2008, Leg. V.I.Gusarov & H.Elven
10029301	*Aloconota currax* (Kraatz, 1856)	Austria, Oberösterreich, Bezirk Kirchdorf an der Krems, 1 km N Molln, Gstadt, Krumme Steyrling, 47°53.676'N 14°15.351'E ±6m, h=383m, flooding river banks, stones, gravel, sand [3312] [Garmin 60CSx; WGS84], 6.v.2008, Leg. V.I.Gusarov & H.Elven
10029294	*Aloconota gregaria* (Erichson, 1839)	Norway, Oslo, Oslo kommune, Kantarellen Terrasse, peninsula, 59°50.481'N 10°46.571'E ±6m (extent=20m), h=9m, sifting leaf litter and moss [3696] [Garmin 60CSx; WGS84], 25.iv.2009, Leg. V.I.Gusarov
10030840	*Aloconota* sp.	Uganda, Western Region, Kasese District, 3.5 km NW Nyakalengija, Rwenzori Mts. National Park, main trail to Nyabitaba Hut, 0°21.679'N 30°00.890'E ±11m (extent=10m), h=1837m, river bank, at the edge of water, sand, stones [3523] [Garmin 60CSx; WGS84], 5.viii.2008, Leg. V.I.Gusarov
10030746	*Aloconota* sp.	Uganda, Western Region, Kasese District, 3.5 km NW Nyakalengija, Rwenzori Mts. National Park, main trail to Nyabitaba Hut, 0°21.679'N 30°00.890'E ±11m (extent=10m), h=1837m, river bank, at the edge of water, sand, stones [3523] [Garmin 60CSx; WGS84], 5.viii.2008, Leg. V.I.Gusarov
10029305	*Alpinia* sp. prope *alpicola* (Miller, 1859)	Romania, jud. Prahova, Munții Bucegi, M-le Babele, 2 km SW Vf. Coştila, 45°25'04"N 25°28'13"E, h=2290m, rocky alpine pasture at path, edge of melting snow patch, at/under stones [217], 28.vi.2006, Leg. Gy.Makranczy
10002644	*Alpinia* sp. prope *alpicola* (Miller, 1859)	Romania, jud. Prahova, Munții Bucegi, M-le Babele, 2 km SW Vf. Coştila, 45°25'04"N 25°28'13"E, h=2290m, rocky alpine pasture, on path, edge of melting snow patch, near/under stones [217], 28.vi.2006, Leg. Gy.Makranczy
10030878*	*Amaurodera yaoana* Pace, 1992	Laos, Champasak prov.: Bolaven plateau, Muang Paxong, Ban Thongvay, 15°14.741'N 106°31.916'E, h=1300m, disturbed primary rainforest; flight intercept trap [LAO08-5a], 9–16.vi.2008, Leg. A.Solodovnikov & J.Pedersen

10030812*	*Amaurodera yaoana* Pace, 1992	Laos, Champasak prov.: Bolaven plateau, Muang Paxong, Ban Thongvay, 15°14.741'N 106°31.916'E, h=1300m, disturbed primary rainforest; flight intercept trap [LAO08-5a], 9–16.vi.2008, Leg. A.Solodovnikov & J.Pedersen
10002646	*Amidobia talpa* (Heer, 1841)	Norway, AK, Oslo: Vettakollen, Båntjern, (UTM) 32V 05952,66487, on anthill (*F. rufa* group) in mixed forest [WGS84], 4.v.2006, Leg. H.Elven
10029292	*Amischa analis* (Gravenhorst, 1802)	Norway, Oslo, Oslo kommune, below Kantarellen Terrasse, 59°50.965'N 10°48.924'E ±4m (extent=20m), h=134m, under stones and sifting dead grass near mice burrows [3693] [Garmin 60CSx; WGS84], 19.iv.2009, Leg. V.I.Gusarov
10002623	*Amischa analis* (Gravenhorst, 1802)	Norway, Akershus, Rælingen, Årnestangen, E bank of the river, 59°53.177'N 11°08.197'E ±8m, h=100m, flood refuse [2356] [Garmin eTrex; WGS84], 7.vi.2006, Leg. V.I.Gusarov
10029304	*Amischa nigrofusca* (Stephens, 1832)	Norway, Oslo, Oslo kommune, below Kantarellen Terrasse, 59°50.965'N 10°48.924'E ±4m (extent=20m), h=134m, under stones and sifting dead grass near mice burrows [3693] [Garmin 60CSx; WGS84], 19.iv.2009, Leg. V.I.Gusarov
10002622	*Amischa nigrofusca* (Stephens, 1832)	Norway, Akershus, Rælingen, Årnestangen, E bank of the river, 59°53.177'N 11°08.197'E ±8m, h=100m, flood refuse [2356] [Garmin eTrex; WGS84], 7.vi.2006, Leg. V.I.Gusarov
10002583	*Atheta aeneipennis* (Thomson, 1856)	Norway, Sør-Trøndelag, Røros kommune, 31 km SE Røros, 3 km NEE Langen, Rundtjønna Lake, W bank, 62°26.584–.489'N 11°54.464–640'E ±14m, h=680m, in fungi [2172] [Garmin eTrex; WGS84], 13.viii.2005, Leg. V.I.Gusarov
10002638	*Atheta bosnica* Ganglbauer, 1895	Romania, jud. Prahova, Munții Bucegi, 0.5 km W Vîrful cu Dor, 45°21'06"N 25°28'56"E, h=1950m, isolated snow patch below summit, in/under grass and stones at the edge of melting snow [213], 27.vi.2006, Leg. Gy.Makranczy
10002560	*Atheta celata* (Erichson, 1837)	Norway, Akershus, Rælingen, Årnestangen, E bank of the river, 59°53.279'N 11°08.062'E ±7m, h=100m, flood refuse on previously flooded field [2357] [Garmin eTrex; WGS84], 7.vi.2006, Leg. V.I.Gusarov
10002556	*Atheta celata* (Erichson, 1837)	Norway, Akershus, Rælingen, Årnestangen, E bank of the river, 59°53.279'N 11°08.062'E ±7m, h=100m, flood refuse on previously flooded field [2357] [Garmin eTrex; WGS84], 7.vi.2006, Leg. V.I.Gusarov
10002582	*Atheta cinnamoptera* (Thomson, 1856)	Norway, env. of Oslo, 2 km SE Skullerud, 59°50.983'N 10°51.263'E ±9m, h=180m, on fungi [2179] [Garmin eTrex; WGS84], 28.viii.2005, Leg. V.I.Gusarov
10002635	*Atheta contristata* (Kraatz, 1856)	Romania, jud. Alba, 1 km NE Scărişoara, Corobana Mîndrutului (cave), entrance zone, 46°27'44"N 22°53'18"E, h=760m, under/around wet stones, on dead insects [224], 30.vi.2006, Leg. Gy.Makranczy

10002640	*Atheta crassicornis* (Fabricius, 1793)	Hungary, Debrecen, Nagyerdő, 47°34'47"N 21°37'36"E, h=500m, oak forest, cheese trap in mole (*Talpa*) nest, 19.vii.2006, Leg. Gy.Makranczy
10002554	*Atheta dadopora* (Thomson, 1867)	U.S.A., Tennessee, Sevier Co., 9.5 km NEE Gatlinburg, Great Smoky Mountains National Park, Greenbrier, nr. entrance, 35°44.265'N 83°24.976'W ±6m, h=455m, in hay [2447] [Garmin eTrex; WGS84], 30.vi.2006, Leg. V.I.Gusarov
10002578	*Atheta gagatina* (Baudi di Selve, 1848)	Norway, env. of Oslo, 2 km SE Skullerud, 59°50.983'N 10°51.263'E ±9m, h=180m, on fungi [2179] [Garmin eTrex; WGS84], 28.viii.2005, Leg. V.I.Gusarov
10002580	*Atheta gagatina* (Baudi di Selve, 1848)	Norway, env. of Oslo, 2 km SE Skullerud, 59°50.983'N 10°51.263'E ±9m, h=180m, on fungi [2179] [Garmin eTrex; WGS84], 28.viii.2005, Leg. V.I.Gusarov
10002561	*Atheta graminicola* (Gravenhorst, 1806)	Norway, Akershus, Rælingen, Årnestangen, E bank of the river, 59°53.177'N 11°08.197'E ±8m, h=100m, flood refuse [2356] [Garmin eTrex; WGS84], 7.vi.2006, Leg. V.I.Gusarov
10002562	*Atheta graminicola* (Gravenhorst, 1806)	Norway, Akershus, Rælingen, Årnestangen, E bank of the river, 59°53.177'N 11°08.197'E ±8m, h=100m, flood refuse [2356] [Garmin eTrex; WGS84], 7.vi.2006, Leg. V.I.Gusarov
10002586	*Atheta kenyamontis* Pace, 1986	Kenya, Central Prov., Mt.Kenya Nat. Park, Naro Moru route above meteostation, 0°10'04.4"S 37°15'03.8"E ±10m, h=3896m, alpine vegetation belt, in rotting leaves of *Cirsium*-like plant [2668] [Garmin eTrex; WGS84], 8.xi.2006, Leg. V.I.Gusarov
10029115	*Atheta laticeps* (Thomson, 1856)	Belarus, Grodno reg., Shchuchin distr., 5 km NNW Zachepichi, Neman Riv., 53°29'20"N 24°57'52"E ±2000m, riverine oak forest [Garmin eTrex,; WGS84], 28.ix.2006, Leg. A.Derunkov
10029116	*Atheta laticeps* (Thomson, 1856)	Belarus, Grodno reg., Shchuchin distr., 5 km NNW Zachepichi, Neman Riv., 53°29'20"N 24°57'52"E ±2000m, riverine oak forest [Garmin eTrex,; WGS84], 28.ix.2006, Leg. A.Derunkov
10002606	*Atheta laticollis* (Stephens, 1832)	Norway, Akershus, Rælingen, Årnestangen, E bank of the river, 59°53.279'N 11°08.062'E ±7m, h=100m, flood refuse on previously flooded field [2357] [Garmin eTrex; WGS84], 7.vi.2006, Leg. V.I.Gusarov
10002653	*Atheta membranata* G.Benick, 1974	France, Pyrénées-Orientales, Mosset–col de Jau, h=1450m, 19.viii.2007, Leg. M.Tronquet
10002621	*Atheta modesta* (Melsheimer, 1844)	U.S.A., North Carolina, Swain Co., 11–13 km NNW Cherokee, Great Smoky Mountains National Park, Thomas Ridge, Thomas Divide Trail, 35°34.581'–33.995'N 83°23.650'–22.881'W ±5m, h=1530–1590m, in fungi [2470] [Garmin eTrex; WGS84], 4.vii.2006, Leg. V.I.Gusarov
10002620	*Atheta modesta* (Melsheimer, 1844)	U.S.A., North Carolina, Swain Co., 11–13 km NNW Cherokee, Great Smoky Mountains National Park, Thomas Ridge, Thomas Divide Trail, 35°34.581'–33.995'N 83°23.650'–22.881'W ±5m, h=1530–1590m, in fungi [2470] [Garmin eTrex; WGS84], 4.vii.2006, Leg. V.I.Gusarov
10002642	*Atheta pasadenae* Bernhauer, 1906	France, Pyrénées-Orientales, Mosset–col de Jau, h=1450m, 19.viii.2007, Leg. M.Tronquet

10002548	*Atheta ravilla* (Erichson, 1839)	Norway, Sør-Trøndelag, Røros kommune, 31 km SE Røros, 3 km NEE Langen, Rundtjønna Lake, NW bank, 62°26.784'N 11°54.583'E ±8m, h=659m, in fungi [2173] [Garmin eTrex; WGS84], 15.viii.2005, Leg. V.I.Gusarov
10002557	*Atheta ravilla* (Erichson, 1839)	Norway, env. of Oslo, 2 km SE Skullerud, 59°50.983'N 10°51.263'E ±9m, h=180m, on fungi [2179] [Garmin eTrex; WGS84], 28.viii.2005, Leg. V.I.Gusarov
10030796	*Atheta scapularis* (C.R.Sahlberg, 1831)	France, Pyrénées-Orientales, forêt de Rabouillet-Boucheville, h=800–1100m, filet de voiture, 6.v.2008, Leg. M.Tronquet
10030807	*Atheta scapularis* (C.R.Sahlberg, 1831)	France, Pyrénées-Orientales, forêt de Rabouillet-Boucheville, h=800–1100m, filet de voiture, 6.v.2008, Leg. M.Tronquet
10002639	*Atheta setigera* (Sharp, 1869)	Romania, jud. Hunedoara, Parcul National Retezat, Bordu Tomii, 0.5 km S Casc. Pietrele, 45°22'59"N 22°52'39"E, h=1840m, in horse dung on path on large rocks [239], 15.vii.2006, Leg. Gy.Makranczy
10002564	*Atheta* sp. ex gr. *lippa*	U.S.A., Washington, Clallam Co., 5 km S Port Angeles, Olympic Nat. Park, Hurricane Ridge Rd., 48°04.389'N 123°25.986'W ±7m, h=370m, in forest litter, *Acer*, *Tuja*, ferns [2159] [Garmin eTrex; WGS84], 8.vii.2005, Leg. V.I.Gusarov
10002655	*Atheta vaga* (Heer, 1839)	France, Pyrénées-Orientales, Mosset–col de Jau, h=1450m, 19.viii.2007, Leg. M.Tronquet
10002613	*Atheta vestita* (Gravenhorst, 1806)	Norway, NSY; Gildeskål: Sund, (UTM) 33W 04586,74401, h=0m, rotting kelp in bottom of sandy bay [WGS84], 18.vii.2006, Leg. H.Elven
10029114	*Atheta vilis* (Erichson, 1837)	Belarus, Grodno reg., Shchuchin distr., 5 km NNW Zachepichi, Neman Riv., 53°29'20"N 24°57'52"E ±2000m, riverine oak forest [Garmin eTrex; WGS84], 28.ix.2006, Leg. A.Derunkov
10002666	*Atheta vilis* (Erichson, 1837)	Belarus, Grodno reg., Shchuchin distr., 5 km NNW Zachepichi, Neman Riv., 53°29'20"N 24°57'52"E ±2000m, riverine oak forest [Garmin eTrex; WGS84], 28.ix.2006, Leg. A.Derunkov
10002596	*Bolitochara pulchra* (Gravenhorst, 1806)	Norway, Sør-Trøndelag, Røros kommune, 31 km SE Røros, 3 km NEE Langen, Rundtjønna Lake, NW bank, 62°26.784'N 11°54.583'E ±8m, h=659m, in fungi [2173] [Garmin eTrex; WGS84], 15.viii.2005, Leg. V.I.Gusarov
10002591	*Bolitochara pulchra* (Gravenhorst, 1806)	Norway, Sør-Trøndelag, Røros kommune, 31 km SE Røros, 3 km NEE Langen, Rundtjønna Lake, NW bank, 62°26.784'N 11°54.583'E ±8m, h=659m, in fungi [2173] [Garmin eTrex; WGS84], 15.viii.2005, Leg. V.I.Gusarov
10002634	*Boreophilia hyperborea* (Brundin, 1940)	Russia, Magadan Reg., Severo-Evenskiy Distr., env. of Evensk, 61°57'N 159°14'E, low hills surrounded by tussock (*Carex*, *Eriophorum*) tundra; in leaf litter: *Alnus*, *Betula nana*, *Salix*, *Pinus pumila*, moss, 27.vii.2007, Leg. A.S.Ryabukhin

10002633	*Boreostiba* sp.	Russia, Magadan Reg., Severo-Evenskiy Distr., env. of Evensk, 61°57'N 159°14'E, low hills surrounded by tussock (*Carex*, *Eriophorum*) tundra; in leaf litter: *Alnus*, *Betula nana*, *Salix*, *Pinus pumila*, moss, 28.vi.2007, Leg. A.S.Ryabukhin
10002667	*Brundinia meridionalis* (Mulsant & Rey, 1853)	Ukraine, Odessa reg., env. of Odessa, Svitle (Svetloye), ultra-violet light 250W, 26.vii.2008, Leg. A.Gontarenko
10030905	*Callicerus obscurus* Gravenhorst, 1802	Denmark, Fyn: nr Ørbæk, Lykkesholm Vandmolle, 55°14'297"N 10°37'328"E ±180m, h=50m, forest at rim of lake, sifted litter, moss & debris, 27.iii.2009, Leg. A.Schomann & J.Pedersen
10030800	*Callicerus obscurus* Gravenhorst, 1802	Denmark, Fyn: nr Ørbæk, Lykkesholm Vandmolle, 55°14'297"N 10°37'328"E ±180m, h=50m, forest at rim of lake, sifted litter, moss & debris, 27.iii.2009, Leg. A.Schomann & J.Pedersen
10002651	*Cordalia obscura* (Gravenhorst, 1802)	Greece, Corfu, env. of Agios Georgios, 39°26.070'N 19°57.263'E ±5m, h=24m, pile of hay in olive grove [2934] [Garmin 60CSx; WGS84], 26.vii.2007, Leg. V.I.Gusarov
10002630	*Dadobia immersa* (Erichson, 1837)	Norway, AK, Oslo: Bygdøy, Huk, (UTM) 32V 0593398,6641668 ±12m, under bark on dead fir [WGS84], 13.ix.2006, Leg. H.Elven & V.I.Gusarov
10002643	*Dalotia coriaria* (Kraatz, 1856)	France, Pyrénées-Orientales, Molitg-les-Bains, 42°39'N 2°23'E, 21.vii.2007, Leg. M.Tronquet
10029112	*Discerota torrentum* (Kiesenwetter,1850)	France, Ariège, Aulus-les-Bains, h=1000m, sprinkled mosses, 8.vi.2009, Leg. M.Tronquet
10029113	*Discerota torrentum* (Kiesenwetter,1850)	France, Ariège, Aulus-les-Bains, h=1000m, sprinkled mosses, 8.vi.2009, Leg. M.Tronquet
10002604	*Drusilla canaliculata* (Fabricius, 1787)	Norway, Akershus, Rælingen, Årnestangen, E bank of the river, 59°53.177'N 11°08.197'E ±8m, h=100m, flood refuse [2356] [Garmin eTrex; WGS84], 7.vi.2006, Leg. V.I.Gusarov
10002601	*Drusilla canaliculata* (Fabricius, 1787)	Norway, Akershus, Rælingen, Årnestangen, E bank of the river, 59°53.279'N 11°08.062'E ±7m, h=100m, flood refuse on previously flooded field [2357] [Garmin eTrex; WGS84], 7.vi.2006, Leg. V.I.Gusarov
10051252	*Drusilla* sp. 1	Thailand, Phetchabun, Khao Kho NP, 16°32.546'N 101°2.501'E, h=560m, mixed deciduous forest, Malaise trap [T1180], 5–12.xii.2006, Leg. Somchai Chachumnan & Saink Singtong
10051251	*Drusilla* sp. 1	Thailand, Phetchabun, Khao Kho NP, 16°32.546'N 101°2.501'E, h=560m, mixed deciduous forest, Malaise trap [T1180], 5–12.xii.2006, Leg. Somchai Chachumnan & Saink Singtong
10051193*	*Drusilla* sp. 2	Laos, Champasak prov.: Bolaven plateau, Muang Paxong, Ban Thongvay, 15°14.741'N 106°31.916'E, h=1300m, disturbed primary rainforest; flight intercept trap [LAO08-5a], 9–16.vi.2008, Leg. A.Solodovnikov & J.Pedersen
10051194*	*Drusilla* sp. 2	Laos, Champasak prov.: Bolaven plateau, Muang Paxong, Ban Thongvay, 15°14.741'N 106°31.916'E, h=1300m, disturbed primary rainforest; flight intercept trap [LAO08-5a], 9–16.vi.2008, Leg. A.Solodovnikov & J.Pedersen

10051166*	*Drusilla* sp. prope *khamhengi* Pace, 1984	Laos, Vientiane prov., Houayang Nat. Park, 18°05.889'N 102°40.521'E, h=180m, mostly secondary rainforest, pitfall traps [LAO08-2f], 3–19.vi.2008, Leg. A.Solodovnikov & J.Pedersen
10051165*	*Drusilla* sp. prope *khamhengi* Pace, 1984	Laos, Vientiane prov., Houayang Nat. Park, 18°05.889'N 102°40.521'E, h=180m, mostly secondary rainforest, pitfall traps [LAO08-2f], 3–19.vi.2008, Leg. A.Solodovnikov & J.Pedersen
10002539	*Earota dentata* (Bernhauer, 1906)	U.S.A., Tennessee, Sevier Co., 11.5 km E Gatlinburg, Great Smoky Mountains National Park, Greenbrier, pavilion, 35°42.219'N 83°23.165'W ±9m, h=575m, light trap [2419] [Garmin eTrex; WGS84], 24.vi.2006, Leg. V.I.Gusarov
10029306	*Earota reyi* (Kiesenwetter, 1850)	France, Pyrénées-Orientales, forêt de Rabouillet-Boucheville, h=800–1100m, filet de voiture, 6.v.2008, Leg. M.Tronquet
10029307	*Earota reyi* (Kiesenwetter, 1850)	France, Pyrénées-Orientales, forêt de Rabouillet-Boucheville, h=800–1100m, filet de voiture, 6.v.2008, Leg. M.Tronquet
10029249	*Ecitodonia* sp.	Ecuador, Orellana, Yasuni Research Station, recently abandoned *Eciton burchelli* bivouac site, 13.vii.2008, Leg. A.K.Tishechkin
10029248	*Ecitodonia* sp.	Ecuador, Orellana, Yasuni Research Station, recently abandoned *Eciton burchelli* bivouac site, 13.vii.2008, Leg. A.K.Tishechkin
10002689	*Ecitomorpha* sp.	Peru, Junín, 11 km NE Puerto Ocopa, Los Olivos, 11°3.00'S 74°15.52'W, h=1200m, Emigr. Column of *Eciton*, colony EP-01, AT1080, 27.iii.2009, Leg. A.K.Tishechkin
10002592	*Ecitophya gracillima* Mann, 1925	Peru, Loreto, 1 km E Jenaro Herrero, IIAP Centro de Investigaciones Jenaro Herrerra, 4°53'36.4"S 73°39'01.6"W ±19m, h=103m, with army ants *Eciton hamatum* (F.) [2593] [Garmin eTrex; WGS84], 26.ix.2006, Leg. V.I.Gusarov
10029164	*Ecitophya gracillima* Mann, 1925	Peru, Loreto, 1 km E Jenaro Herrero, IIAP Centro de Investigaciones Jenaro Herrerra, 4°53'36.4"S 73°39'01.6"W ±19m, h=103m, with army ants *Eciton hamatum* (F.) [2593] [Garmin eTrex; WGS84], 26.ix.2006, Leg. V.I.Gusarov
10029251	*Ecitopora* sp.	Ecuador, Orellana, Yasuni Research Station, recently abandoned *Eciton burchelli* bivouac site, 13.vii.2008, Leg. A.K.Tishechkin
10030875	*Geostiba bicarinata* Lohse & Smetana, 1988	U.S.A., North Carolina, Haywood Co./ Jackson Co., 14 km S Waynesville, Blue Ridge Parkway, Richland Balsam, S slope, 35°21.590'N 82°59.212'W ±9m, h=1840m, in forest litter, *Picea*, *Betula*, ferns, *Oxalis*, *Rubus* [2479] [Garmin eTrex; WGS84], 5.vii.2006, Leg. V.I.Gusarov
10030948	*Geostiba bicarinata* Lohse & Smetana, 1988	U.S.A., North Carolina, Haywood Co./ Jackson Co., 14 km S Waynesville, Blue Ridge Parkway, Richland Balsam, S slope, 35°21.590'N 82°59.212'W ±9m, h=1840m, in forest litter, *Picea*, *Betula*, ferns, *Oxalis*, *Rubus* [2479] [Garmin eTrex; WGS84], 5.vii.2006, Leg. V.I.Gusarov

10002587	*Geostiba circellaris* (Gravenhorst, 1806)	Norway, env. of Oslo, 2 km SE Skullerud, 59°50.983'N 10°51.263'E ±9m, h=180m, in forest litter, *Picea*, *Vaccinium myrt*., *Pinus*, *Betula* [2180] [Garmin eTrex; WGS84], 28.viii.2005, Leg. V.I.Gusarov
10030888	*Geostiba nubigena* Lohse & Smetana, 1988	U.S.A., North Carolina, Haywood Co./ Jackson Co., 14 km S Waynesville, Blue Ridge Parkway, Richland Balsam, S slope, 35°21.590'N 82°59.212'W ±9m, h=1840m, in forest litter, *Picea*, *Betula*, ferns, *Oxalis*, *Rubus* [2479] [Garmin eTrex; WGS84], 5.vii.2006, Leg. V.I.Gusarov
10030736	*Geostiba nubigena* Lohse & Smetana, 1988	U.S.A., North Carolina, Haywood Co./ Jackson Co., 14 km S Waynesville, Blue Ridge Parkway, Richland Balsam, S slope, 35°21.590'N 82°59.212'W ±9m, h=1840m, in forest litter, *Picea*, *Betula*, ferns, *Oxalis*, *Rubus* [2479] [Garmin eTrex; WGS84], 5.vii.2006, Leg. V.I.Gusarov
10002641	*Gymnusa variegata* Kiesenwetter, 1845	Romania, jud. Harghita, 4 km ESE Bǎile Tuşnad, N edge of Tinovul Mohoş, slope/bank of stream Roşu, 46°08'15"N 25°54'27"E, h=1050m, muddy treaded grass on streamlet bank, flotation [206], 11.vi.2006, Leg. Gy.Makranczy
10002584	*Gyrophaena congrua* Erichson, 1837	Norway, env. of Oslo, W bank of Sognsvann Lake, 59°58'19"N 10°43'22"E ±8m, h=200m, in mushrooms (*Kuehneromyces mutabilis*) [2171] [Google Earth; WGS84], 7.viii.2005, Leg. V.I.Gusarov
10002585	*Gyrophaena fasciata* (Marsham, 1802)	Norway, env. of Oslo, W bank of Sognsvann Lake, 59°58'19"N 10°43'22"E ±8m, h=200m, in mushrooms (*Kuehneromyces mutabilis*) [2171] [Google Earth; WGS84], 7.viii.2005, Leg. V.I.Gusarov
10002572	*Gyrophaena fasciata* (Marsham, 1802)	Norway, env. of Oslo, W bank of Sognsvann Lake, 59°58'19"N 10°43'22"E ±8m, h=200m, in mushrooms (*Kuehneromyces mutabilis*) [2171] [Google Earth; WGS84], 7.viii.2005, Leg. V.I.Gusarov
10002647	*Halobrecta* cf. *halensis* Mulsant & Rey, 1873	Greece, Ionian Islands, Is. Lefkada, Lefkadha (Lefkada) City, S coast of enclosed bay N of the city, 38°50.153'N 20°42.556'E ±5m, h=0m, in seaweed pile on beach [2544] [Garmin eTrex; WGS84], 7.vii.2006, Leg. V.I.Gusarov
10002550	*Hoplandria lateralis* (Melsheimer, 1846)	U.S.A., Tennessee, Sevier Co., 11.5 km E Gatlinburg, Great Smoky Mountains National Park, Greenbrier, pavilion, 35°42.219'N 83°23.165'W ±9m, h=575m, light trap [2419] [Garmin eTrex; WGS84], 24.vi.2006, Leg. V.I.Gusarov
10002661	*Hydrosmecta eximia* (Sharp, 1869)	Austria, Oberösterreich, Bezirk Kirchdorf an der Krems, 1 km N Molln, Gstadt, Krumme Steyrling, 47°53.676'N 14°15.351'E ±6m, h=383m, flooding river banks, stones, gravel, sand [3312] [Garmin 60CSx; WGS84], 6.v.2008, Leg. V.I.Gusarov & H.Elven
10002659	*Hydrosmecta eximia* (Sharp, 1869)	Austria, Oberösterreich, Bezirk Kirchdorf an der Krems, 1 km N Molln, Gstadt, Krumme Steyrling, 47°53.676'N 14°15.351'E ±6m, h=383m, flooding river banks, stones, gravel, sand

		[3312] [Garmin 60CSx; WGS84], 6.v.2008, Leg. V.I.Gusarov & H.Elven
10002658	*Hydrosmecta gracilicornis* (Erichson, 1839)	Austria, Oberösterreich, Bezirk Kirchdorf an der Krems, 1 km N Molln, Gstadt, Krumme Steyrling, 47°53.676'N 14°15.351'E ±6m, h=383m, flooding river banks, stones, gravel, sand [3312] [Garmin 60CSx; WGS84], 6.v.2008, Leg. V.I.Gusarov & H.Elven
10002650	*Hydrosmecta* sp. 1	U.S.A., Oregon, Curry Co., 23 km SE Port Orford, NFD 3402, Euchre Creek, 42°35.142'N 124°19.716'W ±8m, h=20m, creek banks, fine gravel [2139] [Garmin eTrex; WGS84], 6.vii.2005, Leg. V.I.Gusarov
10002660	*Hydrosmecta* sp. 2	Austria, Oberösterreich, Bezirk Kirchdorf an der Krems, 1 km N Molln, Gstadt, Krumme Steyrling, 47°53.676'N 14°15.351'E ±6m, h=383m, flooding river banks, stones, gravel, sand [3312] [Garmin 60CSx; WGS84], 6.v.2008, Leg. V.I.Gusarov & H.Elven
10002662	*Hydrosmecta valdieriana* (Scheerpeltz, 1944)	Austria, Oberösterreich, Bezirk Kirchdorf an der Krems, 1 km N Molln, Gstadt, Krumme Steyrling, 47°53.676'N 14°15.351'E ±6m, h=383m, flooding river banks, stones, gravel, sand [3312] [Garmin 60CSx; WGS84], 6.v.2008, Leg. V.I.Gusarov & H.Elven
10002663	*Hydrosmecta valdieriana* (Scheerpeltz, 1944)	Austria, Oberösterreich, Bezirk Kirchdorf an der Krems, 1 km N Molln, Gstadt, Krumme Steyrling, 47°53.676'N 14°15.351'E ±6m, h=383m, flooding river banks, stones, gravel, sand [3312] [Garmin 60CSx; WGS84], 6.v.2008, Leg. V.I.Gusarov & H.Elven
10002600	*Liogluta microptera* Thomson, 1867	Czech Republic, Jihočeský Region, 50 km SWW České Budějovice, Šumava National Park, 48°51.552'N 13°49.104'E ±5m, h=780m, right bank of the river, in flood refuse [2348] [Garmin eTrex; WGS84], 28.v.2006, Leg. V.I.Gusarov
10002602	*Liogluta microptera* Thomson, 1867	Czech Republic, Jihočeský Region, 50 km SWW České Budějovice, Šumava National Park, 48°51.552'N 13°49.104'E ±5m, h=780m, right bank of the river, in flood refuse [2348] [Garmin eTrex; WGS84], 28.v.2006, Leg. V.I.Gusarov
10002636	*Liogluta nigropolita* (Bernhauer, 1907)	Russia, Magadan Reg., Severo-Evenskiy Distr., env. of Evensk, 61°57'N 159°14'E, low hills surrounded by tussock (Carex, Eriophorum) tundra; in leaf litter: *Alnus*, *Betula nana*, *Salix*, *Pinus pumila*, moss, 27.vii.2007, Leg. A.S.Ryabukhin
10030941	*Lomechusa emarginata* (Paykull, 1789)	Norway, EIS 28 AK, ASKER: Brønnøya, Store Ostsundet, (UTM) NM 87013,37111 ±8m, h=10m, *Myrmica* nest under stone. SE faced slope, clay stone [HE09-12D] [WGS84], 15.v.2009, Leg. H.Elven
10030947	*Lomechusa emarginata* (Paykull, 1789)	Norway, EIS 28 AK, ASKER: Brønnøya, Store Ostsundet, (UTM) NM 87009,37108 ±6m, h=10m, *Myrmica* nest under stone. SE faced slope, clay stone [HE09-12C] [WGS84], 15.v.2009, Leg. H.Elven
10030917	*Lomechusa pubicollis* Brisout de Barneville, 1860	Germany, Mark Brandenburg, Müncheberg, 52°32.016'N 14°01.665'E ±5m (extent=5m), h=44m,

		in *Formica* nest (hill) [3750] [Garmin 60CSx; WGS84], 23.v.2009, Leg. V.I.Gusarov
10029159	Lomechusini genus 1	Ecuador, Orellana, Yasuni Research Station, recently abandoned *Eciton burchelli* bivouac site, 13.vii.2008, Leg. A.K.Tishechkin
10002685	Lomechusini genus 2	Ecuador, Orellana, Yasuni Research Station, recently abandoned *Eciton burchelli* bivouac site, 13.vii.2008, Leg. A.K.Tishechkin
10029161	Lomechusini genus 2	Ecuador, Orellana, Yasuni Research Station, recently abandoned *Eciton burchelli* bivouac site, 13.vii.2008, Leg. A.K.Tishechkin
10029252	Lomechusini genus 3	Ecuador, Orellana, Yasuni Research Station, recently abandoned *Eciton burchelli* bivouac site, 13.vii.2008, Leg. A.K.Tishechkin
10029253	Lomechusini genus 3	Ecuador, Orellana, Yasuni Research Station, recently abandoned *Eciton burchelli* bivouac site, 13.vii.2008, Leg. A.K.Tishechkin
10029256	Lomechusini genus 4	Ecuador, Orellana, Yasuni Research Station, recently abandoned *Eciton burchelli* bivouac site, 13.vii.2008, Leg. A.K.Tishechkin
10029254	Lomechusini genus 4	Ecuador, Orellana, Yasuni Research Station, recently abandoned *Eciton burchelli* bivouac site, 13.vii.2008, Leg. A.K.Tishechkin
10029258	Lomechusini genus 5	Ecuador, Orellana, Yasuni Research Station, recently abandoned *Eciton burchelli* bivouac site, 13.vii.2008, Leg. A.K.Tishechkin
10029257	Lomechusini genus 5	Ecuador, Orellana, Yasuni Research Station, recently abandoned *Eciton burchelli* bivouac site, 13.vii.2008, Leg. A.K.Tishechkin
10029279	Lomechusini genus 6	Ecuador, Orellana, Yasuni Research Station, recently abandoned *Eciton burchelli* bivouac site, 13.vii.2008, Leg. A.K.Tishechkin
10029280	Lomechusini genus 7	Ecuador, Orellana, Yasuni Research Station, recently abandoned *Eciton burchelli* bivouac site, 13.vii.2008, Leg. A.K.Tishechkin
10029282	Lomechusini genus 7	Ecuador, Orellana, Yasuni Research Station, recently abandoned *Eciton burchelli* bivouac site, 13.vii.2008, Leg. A.K.Tishechkin
10030868	*Lomechusoides amurensis* (Wasmann, 1897)	Russia, Irkutsk Reg., Irkutsk, right bank of the Irkut River, crumbly stump with ants, 28.iv.2009, Leg. I.Enustschenko
10002632	*Lypoglossa lateralis* (Mannerheim, 1830)	Russia, Magadan Reg., Severo-Evenskiy Distr., env. of Evensk, 61°57'N 159°14'E, low hills surrounded by tussock (Carex, Eriophorum) tundra; in leaf litter: *Alnus*, *Betula nana*, *Salix*, *Pinus pumila*, moss, 28.vi.2007, Leg. A.S.Ryabukhin
10002649	*Lyprocorrhe anceps* (Erichson, 1837)	Norway, AK, Oslo: Vettakollen, Båntjern, (UTM) 32V 05952,66487, on anthill (*F. rufa* group) in

		mixed forest [WGS84], 4.v.2006, Leg. H.Elven
10002576	*Meronera venustula* (Erichson, 1839)	U.S.A., Kansas, Douglas Co., Lawrence, 38°59.59'N 95°14.59'W, h=200m, in flood refuse (mostly rotting old corn stems) [2050] [Garmin eTrex; WGS84], 19.vi.2005, Leg. V.I.Gusarov
10002588	*Mocyta fungi* (Gravenhorst, 1806)	Germany, Brandenburg, "Märkische Schweiz", nature reserve "Großer Klobichsee" W Münchehofe, 52°33.526'N 14°07.584'E ±13m, h=30m, in leaf litter, *Quercus*, *Alnus*, *Galium*, *Glechoma*, *Cardamine*, *Primula*, *Corydalis* [2028] [Garmin eTrex; WGS84], 7.v.2005, Leg. V.I.Gusarov
10002589	*Mocyta fungi* (Gravenhorst, 1806)	Germany, Brandenburg, "Märkische Schweiz", nature reserve "Großer Klobichsee" W Münchehofe, 52°33.526'N 14°07.584'E ±13m, h=30m, in leaf litter, *Quercus*, *Alnus*, *Galium*, *Glechoma*, *Cardamine*, *Primula*, *Corydalis* [2028] [Garmin eTrex; WGS84], 7.v.2005, Leg. V.I.Gusarov
10002540	*Mocyta scopula* (Casey, 1893)	U.S.A., Kansas, Douglas Co., 18 km SSE Lawrence, Breidenthal Preserve, 38°48.589'N 95°11.409'W ±7m, h=260m, in forest litter, *Quercus* [2044] [Garmin eTrex; WGS84], 18.vi.2005, Leg. V.I.Gusarov
10002559	*Mocyta scopula* (Casey, 1893)	U.S.A., Kansas, Douglas Co., 18 km SSE Lawrence, Breidenthal Preserve, 38°48.589'N 95°11.409'W ±7m, h=260m, in forest litter, *Quercus* [2044] [Garmin eTrex; WGS84], 18.vi.2005, Leg. V.I.Gusarov
10030903	*Myllaena audax* Casey, 1911	U.S.A., Oregon, Coos Co., 6 km N Coos Bay, Oregon Dunes Nat. Rec. Area, Siuslaw Nat. Forest, 43°26.908'N 124°14.945'W ±7m, h=0m, lake banks, *Carex*, organic debris [2143] [Garmin eTrex; WGS84], 6.vii.2005, Leg. V.I.Gusarov
10002598	*Myllaena audax* Casey, 1911	U.S.A., Oregon, Coos Co., 6 km N Coos Bay, Oregon Dunes Nat. Rec. Area, Siuslaw Nat. Forest, 43°26.908'N 124°14.945'W ±7m, h=0m, lake banks, *Carex*, organic debris [2143] [Garmin eTrex; WGS84], 6.vii.2005, Leg. V.I.Gusarov
10030945	*Myrmecopora uvida* (Erichson, 1840)	Greece, Ionian Islands, Is. Lefkada, Lefkadha (Lefkada) City, S coast of enclosed bay N of the city, 38°50.153'N 20°42.556'E ±5m, h=0m, in seaweed pile on beach [2544] [Garmin eTrex; WGS84], 7.vii.2006, Leg. V.I.Gusarov
10029111	*Myrmecopora uvida* (Erichson, 1840)	Greece, Ionian Islands, Is. Lefkada, Lefkadha (Lefkada) City, S coast of enclosed bay N of the city, 38°50.153'N 20°42.556'E ±5m, h=0m, in seaweed pile on beach [2544] [Garmin eTrex; WGS84], 7.vii.2006, Leg. V.I.Gusarov
10002615	*Myrmedonota* sp.	U.S.A., Tennessee, Sevier Co., 11 km SEE Gatlinburg, Great Smoky Mountains National Park, Porters Creek trail, 35°40.928'N 83°23.810'W ±11m, h=775m, on fermenting oak sap [2433] [Garmin eTrex; WGS84], 27.vi.2006, Leg. V.I.Gusarov

10002614	*Myrmedonota* sp.	U.S.A., Tennessee, Sevier Co., 11.5 km E Gatlinburg, Great Smoky Mountains National Park, Greenbrier, pavilion, 35°42.219'N 83°23.165'W ±9m, h=575m, light trap [2419] [Garmin eTrex; WGS84], 24.vi.2006, Leg. V.I.Gusarov
10051237*	*Orphnebius* sp. 1	Laos, Vientiane prov., Phou Khao Khouay, 18°20.369'N 102°48.523'E, h=700–800m, strongly disturbed primary rainforest, nr. forest edge, flight intercept trap [LAO08-1j], 26–31.v.2008, Leg. A.Solodovnikov & J.Pedersen
10051236*	*Orphnebius* sp. 2	Laos, Champasak prov., Bolaven plateau, Muang Paxong, Ban Thongvay, 15°14.054'N 106°31.867'E, h=1200m, edge of disturbed primary rainforest (nr. clearing), Malaise trap [LAO08-6b], 8–16.vi.2008, Leg. A.Solodovnikov & J.Pedersen
10051239	*Orphnebius* sp. 3	Thailand, Phetchabun, Thung Salaeng Luang NP, Gang wang nam yen, 16°35.789'N 100°52.286'E, h=769m, Malaise trap, pine forest [T1291], 4–11.i.2007, Leg. Pongpitak & Sathit
10051238	*Orphnebius* sp. 4	Thailand, Phetchabun, Thung Salaeng Luang NP, Gang wang nam yen, 16°35.789'N 100°52.286'E, h=769m, Malaise trap, pine forest [T1291], 4–11.i.2007, Leg. Pongpitak & Sathit
10002637	*Oxypoda praecox* Erichson, 1839	Germany, Brandenburg, “Märkische Schweiz”, nature reserve “Großer Klobichsee” W Münchehofe, 52°33.765'N 14°07.415'E ±13m, h=40m, in leaf litter, *Betula*, *Alnus*, *Carex*, *Urtica*, in wet hollow [2025] [Garmin eTrex; WGS84], 7.v.2005, Leg. V.I.Gusarov
10051235	*Pedinopleurus* sp.	Thailand, Phetchabun, Khao Kho NP, 16°32.546'N 101°2.501'E, h=560m, mixed deciduous forest, Malaise trap [T1189], 26.xii.2006–2.i.2007, Leg. Somchai Chachumnan & Saink Singtong
10030959*	*Pelioptera* sp. prope *micans* (Kraatz, 1857)	Laos, Champasak prov.: Bolaven plateau, Muang Paxong, Ban Thongvay, 15°14.741'N 106°31.916'E, h=1300m, disturbed primary rainforest; flight intercept trap [LAO08-5a], 9–16.vi.2008, Leg. A.Solodovnikov & J.Pedersen
10002617	*Pella caliginosa* (Casey, 1893)	U.S.A., Tennessee, Sevier Co., 11.5 km E Gatlinburg, Great Smoky Mountains National Park, Greenbrier, pavilion, 35°42.219'N 83°23.165'W ±9m, h=575m, light trap [2419] [Garmin eTrex; WGS84], 24.vi.2006, Leg. V.I.Gusarov
10002616	*Pella caliginosa* (Casey, 1893)	U.S.A., Tennessee, Sevier Co., 11.5 km E Gatlinburg, Great Smoky Mountains National Park, Greenbrier, pavilion, 35°42.219'N 83°23.165'W ±9m, h=575m, light trap [2419] [Garmin eTrex; WGS84], 24.vi.2006, Leg. V.I.Gusarov
10002569	*Pella humeralis* (Gravenhorst, 1802)	Norway, env. of Oslo, 1.5 km SE Skullerud, 59°51.271'N 10°51.538'E ±10m, h=200m, in forest litter, *Picea*, *Betula*, *Populus tremula* [2030] [Garmin eTrex; WGS84], 15.v.2005, Leg. V.I.Gusarov
10002607	*Philhygra debilis* (Erichson, 1837)	Norway, Akershus, Rælingen, Årnestangen, E bank of the river, 59°53.177'N 11°08.197'E ±8m, h=100m, flood refuse [2356] [Garmin eTrex; WGS84], 7.vi.2006, Leg. V.I.Gusarov
10002608	*Philhygra debilis* (Erichson, 1837)	Norway, Akershus, Rælingen, Årnestangen, E bank of the river, 59°53.177'N 11°08.197'E ±8m,

		h=100m, flood refuse [2356] [Garmin eTrex; WGS84], 7.vi.2006, Leg. V.I.Gusarov
10002610	*Philhygra fallaciosa* (Sharp, 1869)	Czech Republic, Jihočeský Region, 50 km SWW České Budějovice, Šumava National Park, 48°51.552'N 13°49.104'E ±5m, h=780m, right bank of the river, in flood refuse [2348] [Garmin eTrex; WGS84], 28.v.2006, Leg. V.I.Gusarov
10002609	*Philhygra fallaciosa* (Sharp, 1869)	Czech Republic, Jihočeský Region, 50 km SWW České Budějovice, Šumava National Park, 48°51.552'N 13°49.104'E ±5m, h=780m, right bank of the river, in flood refuse [2348] [Garmin eTrex; WGS84], 28.v.2006, Leg. V.I.Gusarov
10002595	*Philhygra iterans* (Casey, 1910)	U.S.A., Tennessee, Sevier Co., 11.5 km E Gatlinburg, Great Smoky Mountains National Park, Greenbrier, pavilion, 35°42.219'N 83°23.165'W ±9m, h=575m, light trap [2419] [Garmin eTrex; WGS84], 24.vi.2006, Leg. V.I.Gusarov
10002594	*Philhygra iterans* (Casey, 1910)	U.S.A., Tennessee, Sevier Co., 11.5 km E Gatlinburg, Great Smoky Mountains National Park, Greenbrier, pavilion, 35°42.219'N 83°23.165'W ±9m, h=575m, light trap [2419] [Garmin eTrex; WGS84], 24.vi.2006, Leg. V.I.Gusarov
10002541	*Placusa* sp. prope *tachyporoides* (Waltl, 1838)	U.S.A., Tennessee, Sevier Co., 11 km SEE Gatlinburg, Great Smoky Mountains National Park, Porters Creek trail, 35°40.928'N 83°23.810'W ±11m, h=775m, on fermenting oak sap [2433] [Garmin eTrex; WGS84], 27.vi.2006, Leg. V.I.Gusarov
10002577	*Pontomalota opaca* (LeConte, 1863)	U.S.A., Washington, Jefferson Co., 3 km S Kalaloch, Olympic Nat. Park, 47°34.734'N 124°21.903'W ±8m, h=0m, ocean beach, on sand under seaweed [2145] [Garmin eTrex; WGS84], 7.vii.2005, Leg. V.I.Gusarov
10002574	*Pontomalota opaca* (LeConte, 1863)	U.S.A., Washington, Jefferson Co., 3 km S Kalaloch, Olympic Nat. Park, 47°34.734'N 124°21.903'W ±8m, h=0m, ocean beach, on sand under seaweed [2145] [Garmin eTrex; WGS84], 7.vii.2005, Leg. V.I.Gusarov
10002625	*Silusida marginella* (Casey, 1893)	U.S.A., North Carolina, Swain Co., 11–13 km NNW Cherokee, Great Smoky Mountains National Park, Thomas Ridge, Thomas Divide Trail, 35°34.581'–33.995'N 83°23.650'–22.881'W ±5m, h=1530–1590m, in fungi [2470] [Garmin eTrex; WGS84], 4.vii.2006, Leg. V.I.Gusarov
10002624	*Silusida marginella* (Casey, 1893)	U.S.A., North Carolina, Swain Co., 11–13 km NNW Cherokee, Great Smoky Mountains National Park, Thomas Ridge, Thomas Divide Trail, 35°34.581'–33.995'N 83°23.650'–22.881'W ±5m, h=1530–1590m, in fungi [2470] [Garmin eTrex; WGS84], 4.vii.2006, Leg. V.I.Gusarov
10002567	*Stethusa dichroa* (Gravenhorst, 1802)	U.S.A., Tennessee, Sevier Co., 11.5 km E Gatlinburg, Great Smoky Mountains National Park, Greenbrier, pavilion, 35°42.219'N 83°23.165'W ±9m, h=575m, light trap [2419] [Garmin eTrex; WGS84], 24.vi.2006, Leg. V.I.Gusarov
10002568	*Stethusa dichroa* (Gravenhorst, 1802)	U.S.A., Tennessee, Sevier Co., 11.5 km E Gatlinburg, Great Smoky Mountains National Park,

		Greenbrier, pavilion, 35°42.219'N 83°23.165'W ±9m, h=575m, light trap [2419] [Garmin eTrex; WGS84], 24.vi.2006, Leg. V.I.Gusarov
10002599	*Stethusa spuriella* (Casey, 1910)	U.S.A., Tennessee, Sevier Co., 9.5 km NEE Gatlinburg, Great Smoky Mountains National Park, Greenbrier, nr. entrance, 35°44.265'N 83°24.976'W ±6m, h=455m, in hay [2447] [Garmin eTrex; WGS84], 30.vi.2006, Leg. V.I.Gusarov
10002628	*Stethusa spuriella* (Casey, 1910)	U.S.A., Tennessee, Sevier Co., 9.5 km NEE Gatlinburg, Great Smoky Mountains National Park, Greenbrier, nr. entrance, 35°44.265'N 83°24.976'W ±6m, h=455m, in hay [2447] [Garmin eTrex; WGS84], 30.vi.2006, Leg. V.I.Gusarov
10002571	*Strigota ambigua* (Erichson, 1839)	U.S.A., Kansas, Douglas Co., Lawrence, 38°59.59'N 95°14.59'W, h=200m, in flood refuse (mostly rotting old corn stems) [2050] [Garmin eTrex; WGS84], 19.vi.2005, Leg. V.I.Gusarov
10002575	*Strigota ambigua* (Erichson, 1839)	U.S.A., Kansas, Douglas Co., Lawrence, 38°59.075'N 95°14.608'W ±20m, h=200m, edge of flooded field, old corn stems and wet soil [2033] [Garmin eTrex; WGS84], 15.vi.2005, Leg. V.I.Gusarov
10002542	*Tachinus proximus* Kraatz, 1855	Norway, Sør-Trøndelag, Røros kommune, 31 km SE Røros, 3 km NEE Langen, Rundtjønna Lake, W bank, 62°26.584–.489'N 11°54.464–640'E ±14m, h=680m, in fungi [2172] [Garmin eTrex; WGS84], 13.viii.2005, Leg. V.I.Gusarov
10002593	*Tarphiota fucicola* (Mäklin *in* Mannerheim, 1852)	U.S.A., California, Sonoma Co., 21 km SWW Sebastopol, N shore of Bodega Bay, Doran County Park, 38°18.823'N 123°02.384'W ±5m, h=0m, under seaweeds and in sand [2107] [Garmin eTrex; WGS84], 2.vii.2005, Leg. V.I.Gusarov
10029163	*Tetradonia* sp. 1	Ecuador, Orellana, Yasuni Research Station, *Eciton hamatum* emigration column, 22.vii.2008, Leg. A.K.Tishechkin
10029160	*Tetradonia* sp. 2	Ecuador, Orellana, Yasuni Research Station, *Eciton hamatum* emigration column, 22.vii.2008, Leg. A.K.Tishechkin
10029285*	*Tetrasticta* sp. 1	Laos, Champasak prov.: Bolaven plateau, Muang Paxong, Ban Thongvay, 15°14.741'N 106°31.916'E, h=1300m, disturbed primary rainforest; flight intercept trap [LAO08-5a], 9–16.vi.2008, Leg. A.Solodovnikov & J.Pedersen
10029284*	*Tetrasticta* sp. 2	Laos, Champasak prov.: Bolaven plateau, Muang Paxong, Ban Thongvay, 15°14.741'N 106°31.916'E, h=1300m, disturbed primary rainforest; flight intercept trap [LAO08-5a], 9–16.vi.2008, Leg. A.Solodovnikov & J.Pedersen
10002612	*Thendelecrotona* sp.	South Africa, Western Cape, env. of Cape Town, Table Mountain, 33°57.784'S 18°24.396'E ±6m, h=1020m, in *Protea* flowers [2332] [Garmin eTrex; WGS84], 6.iv.2006, Leg. V.I.Gusarov
10002669	*Zyras collaris* (Paykull, 1800)	Abkhasia, left bank of Kodori Riv., nr Naa Village, 42°55'28.6"N E41°17'43.4"E, h=195m, forest

		with *Quercus iberica*, *Carpinus*, *Ruscus ponticus*, *Rhododendron ponticum*, in leaf litter [AB-31], 29.vii.2009, Leg. N.Yunakov
10051274	*Zyras perdecoratus* Pace, 2005	Thailand, Phetchabun, Khao Kho NP, 16°32.546'N 101°2.501'E, h=560m, mixed deciduous forest, Malaise trap [T1189], 26.xii.2006–2.i.2007, Leg. Somchai Chachumnan & Saink Singtong
10051273	*Zyras perdecoratus* Pace, 2005	Thailand, Phetchabun, Khao Kho NP, 16°32.546'N 101°2.501'E, h=560m, mixed deciduous forest, Malaise trap [T1189], 26.xii.2006–2.i.2007, Leg. Somchai Chachumnan & Saink Singtong
10051276	*Zyras* sp. prope *perdecoratus* Pace, 2005	Thailand, Phetchabun, Khao Kho NP, 16°32.546'N 101°2.501'E, h=560m, mixed deciduous forest, Malaise trap [T1189], 26.xii.2006–2.i.2007, Leg. Somchai Chachumnan & Saink Singtong
10051275	*Zyras* sp. prope *perdecoratus* Pace, 2005	Thailand, Phetchabun, Khao Kho NP, 16°32.546'N 101°2.501'E, h=560m, mixed deciduous forest, Malaise trap [T1189], 26.xii.2006–2.i.2007, Leg. Somchai Chachumnan & Saink Singtong
10030963	*Zyras* sp. 1	Thailand, Sakon Nakhon, Phu Phan NP, behind forest protection unit at Huay Wien Prai, 17°6.81'N 104°0.318'E, h=318m, Malaise trap [T1690], 17–25.ii.2007, Leg. Sailom Tongboonchai
10030758	*Zyras* sp. 1	Thailand, Sakon Nakhon, Phu Phan NP, behind forest protection unit at Huay Wien Prai, 17°6.81'N 104°0.318'E, h=318m, Malaise trap [T1690], 17–25.ii.2007, Leg. Sailom Tongboonchai
10051233*	*Zyras* sp. 2	Laos, Vientiane prov., Phou Khao Khouay, 18°20.369'N 102°48.523'E, h=700–800m, strongly disturbed primary rainforest, nr. forest edge, flight intercept trap [LAO08-1j], 26–31.v.2008, Leg. A.Solodovnikov & J.Pedersen
10051234*	*Zyras* sp. 2	Laos, Vientiane prov., Phou Khao Khouay, 18°20.369'N 102°48.523'E, h=700–800m, strongly disturbed primary rainforest, nr. forest edge, flight intercept trap [LAO08-1j], 26–31.v.2008, Leg. A.Solodovnikov & J.Pedersen
10051280	*Zyras* sp. 3	Thailand, Phetchabun, Thung Salaeng Luang NP, Gang wang nam yen, 16°35.789'N 100°52.286'E, h=769m, Malaise trap, pine forest [T1291], 4–11.i.2007, Leg. Pongpitak & Sathit
10051279	*Zyras* sp. 3	Thailand, Phetchabun, Thung Salaeng Luang NP, Gang wang nam yen, 16°35.789'N 100°52.286'E, h=769m, Malaise trap, pine forest [T1291], 4–11.i.2007, Leg. Pongpitak & Sathit
