# Supplementary material for: Molecular phylogeny of the Athetini–Lomechusini–Ecitocharini clade of aleocharine rove beetles (Insecta)
Source: Zool Scr. 2012 Jun 20;41(6):617–36. doi: 10.1111/j.1463-6409.2012.00553.x (PMC3532658; doi:10.1111/j.1463-6409.2012.00553.x)
Supplement: Table S3 — Alignment statistics on the molecular markers used in the study. The four genes COI, COII, 16S and 18S account for 94% of the total alignment, or 3568 sites. [file zsc0041-0617-SD5.pdf]

Supplementary Table S2 Primers used for amplification and sequencing. Alternative primers used to amplify certain difficult samples are indicated with 'alt'. Primers used exclusively as internal sequencing primers are indicated with 'int'.

Target fragment	Ta	Primer	Direction	Primer site	Sequence	Reference
COI (5'-end)	45°C	LCO1490	F	COI	GGT CAA CAA ATC ATA AAG ATA TTG G	Folmer *et al.* 1994[1]
		TY-J-1460	F (alt)	Tyr	TAC AAT TTA TCG CCT AAA CTT CAG CC	Simon *et al.* 1994[2]
		C1-2416ra	R	COI	GCT AAT CAT CTA AAA ATT TTA ATT CC	Modification of C1-J-2441 (Simon *et al.* 1994)
COI (3'-end)	56°C	C1-J-2183	F	COI	CAA CAT TTA TTT TGA TTT TTT GG	Simon *et al.* 1994
		TL2-N-3014	R	Leu2	TCC AAT GCA CTA ATC TGC CAT ATT A	Simon *et al.* 1994
Leu2 + COII	49°C	C1-2441 fa	F	COI	CCT ACA GGA ATT AAA ATT TTT AGT TGA TAA GC	Modification of C1-J-2441 (Simon *et al.* 1994)
		C2-3045 fa	F (int)	Leu2	CAG ATT AGT GCA ATG GAT TTA AGC	Modification of TL2-J-3037 (Simon *et al.* 1994)
		C2-3772 ra	R	COII	GAG ACC ATT ACT TAC TTT CAG CCA TCT	Modification of TK-N-3785 (Simon *et al.* 1994)
		C2-N-3661	R (alt)	COII	CCA CAA ATT TCT GAA CAT TGA CCA	Simon *et al.* 1994
16S + Leu1 + NADH1	51-53°C	LR-N-13398	F	16S	CGC CTG TTT AAC AAA AAC AT	Simon *et al.* 1994
		LR-N-13398c	F (alt)	16S	CGC CTG TTT ATT AAA AAC AT	Modification of LR-N-13398 (Simon *et al.* 1994)
		LR-N-13397	F (alt)	16S	CGC CTG TTT ATC AAA AAC ATS	Modification of LR-N-13398 (Simon *et al.* 1994)
		N1-J-12585	R	NADH1	GGT CCC TTA CGA ATT TGA ATA TAT CCT	Simon *et al.* 1994
		N1-J-12584a	R (alt)	NADH1	GGT CCC TTA CGA ATT TGA ATA TAT CC	Modification of N1-J-12585 (Simon *et al.* 1994)
		N1-J-12530a	R (alt)	NADH1	GCA TCT CTA AAA GGT TG	New
		N1-J-12530b	R (alt)	NADH1	TTA ATA GCA TCT CTA AAA GGT TG	New
18S	58°C	18s ai	F	18S	CCT GAG AAA CGG CTA CCA CAT C	Whiting *et al.* 1997[3]
		18s bi	R	18S	GAG TCT CGT TCG TTA TCG GA	Whiting *et al.* 1997

1) Folmer, O., Black, M., Hoeh, W., Lutz, R. & Vrijenhoek, R. (1994). DNA primers for amplification of mitochondrial cytochrome c oxidase subunit I from diverse metazoan invertebrates. *Molecular Marine Biology and Biotechnology*, 3, 294–299.
2) Simon, C., Frati, F., Beckenbach, A., Crespi, B., Liu, H. & Flook, P. (1994). Evolution, Weighting, and Phylogenetic Utility of Mitochondrial Gene Sequences and a Compilation of Conserved Polymerase Chain Reaction Primers. *Annals of the Entomological Society of America*, 87, 651–701.
3) Whiting, M. F., Carpenter, J. C., Wheeler, Q. D. & Wheeler, W. C. (1997). The Strepsiptera Problem: Phylogeny of the Holometabolous Insect Orders Inferred from 18S and 28S Ribosomal DNA Sequences and Morphology. *Systematic Biology*, 46, 1–68.
