# Supplementary material for: Molecular phylogeny of the Athetini–Lomechusini–Ecitocharini clade of aleocharine rove beetles (Insecta)
Source: Zool Scr. 2012 Jun 20;41(6):617–36. doi: 10.1111/j.1463-6409.2012.00553.x (PMC3532658; doi:10.1111/j.1463-6409.2012.00553.x)
Supplement: Data S1 — ALE_alignment.nex. Sequence alignment in nexus format. Concatenated alignment of two mitochondrial and one nuclear region, covering 3568 bases for 180 samples. [file zsc0041-0617-SD6.pdf]

Supplementary Table S3 Alignment statistics on the molecular markers used in the study. The four genes COI, COII, 16S and 18S account for 94% of the total alignment, or 3568 sites.

Gene	Length	Partition	Sites				
			Excluded	Included			TOTAL
				Constant	Variable		
					Parsimony uninformative	Parsimony informative	
16S	650	Paired sites	-	139	17	93	249
		Unpaired sites	160	146	14	81	401
Leu1	71	Paired sites	-	22	4	12	38
		Unpaired sites	10	16	-	7	33
NADH1	54	1st codon positions	-	1	1	16	18
		2nd codon positions	-	8	1	9	18
		3rd codon positions	-	2	-	16	18
18S	944	Paired sites	38	334	8	80	460
		Unpaired sites	42	329	11	102	484
COI	1461	1st codon positions	1	297	15	174	487
		2nd codon positions	1	409	14	63	487
		3rd codon positions	1	8	5	473	487
COI-Leu2 boundary	10	Undefined	10	-	-	-	10
Leu2	83	Paired sites	-	22	5	13	40
		Unpaired sites	21	13	-	9	43
COII	513	1st codon positions	-	79	13	79	171
		2nd codon positions	-	123	9	39	171
		3rd codon positions	-	4	6	161	171
TOTAL:			284	1952	123	1427	3786
